# Cerebrospinal fluid markers of inflammation and infections in schizophrenia and affective disorders: a systematic review and meta-analysis

**DOI:** 10.1038/s41380-018-0220-4

**Published:** 2018-08-16

**Authors:** Sonja Orlovska-Waast, Ole Köhler-Forsberg, Sophie Wiben Brix, Merete Nordentoft, Daniel Kondziella, Jesper Krogh, Michael Eriksen Benros

**Affiliations:** 1 Mental Health Centre Copenhagen, University of Copenhagen, Faculty of Health Sciences, Copenhagen, Denmark; 2 iPSYCH The Lundbeck Foundation Initiative for Integrative Psychiatric Research, Aarhus, Denmark; 3 Psychosis Research Unit, Aarhus University Hospital, Risskov, Denmark; 4 Department of Clinical Medicine, Aarhus University, Aarhus, Denmark; 5 Department of Neurology, Rigshospitalet, Copenhagen University Hospital, Copenhagen, Denmark; 6 Department of Neuroscience, Norwegian University of Science and Technology, Trondheim, Norway

**Keywords:** Diagnostic markers, Schizophrenia, Depression

## Abstract

Infections and inflammatory processes have been associated with the development of schizophrenia and affective disorders; however, no study has yet systematically reviewed all available studies on cerebrospinal fluid (CSF) immune alterations. We aimed to systematically review the CSF immunological findings in schizophrenia spectrum and affective disorders. We identified all studies investigating CSF inflammatory markers in persons with schizophrenia or affective disorders published prior to March 23, 2017 searching PubMed, CENTRAL, EMBASE, Psychinfo, and LILACS. Literature search, data extraction and bias assessment were performed by two independent reviewers. Meta-analyses with standardized mean difference (SMD) including 95% confidence intervals (CI) were performed on case-healthy control studies. We identified 112 CSF studies published between 1942–2016, and 32 case-healthy control studies could be included in meta-analyses. Studies varied regarding gender distribution, age, disease duration, treatment, investigated biomarkers, and whether recruitment happened consecutively or based on clinical indication. The CSF/serum albumin ratio was increased in schizophrenia (1 study [54 patients]; SMD = 0.71; 95% CI 0.33–1.09) and affective disorders (4 studies [298 patients]; SMD = 0.41; 95% CI 0.23–0.60, *I*^2^ = 0%), compared to healthy controls. Total CSF protein was elevated in both schizophrenia (3 studies [97 patients]; SMD = 0.41; 95% CI 0.15–0.67, *I*^2^ = 0%) and affective disorders (2 studies [53 patients]; SMD = 0.80; 95% CI 0.39–1.21, *I*^2^ = 0%). The IgG ratio was increased in schizophrenia (1 study [54 patients]; SMD = 0.68; 95% CI 0.30–1.06), whereas the IgG Albumin ratio was decreased (1 study [32 patients]; SMD = −0.62; 95% CI −1.13 to −0.12). Interleukin-6 (IL-6) levels (7 studies [230 patients]; SMD = 0.55; 95% CI 0.35–0.76; *I*^2^ = 1%) and IL-8 levels (3 studies [95 patients]; SMD = 0.46; 95% CI 0.17–0.75, *I*^2^ = 0%) were increased in schizophrenia but not significantly increased in affective disorders. Most of the remaining inflammatory markers were not significantly different compared to healthy controls in the meta-analyses. However, in the studies which did not include healthy controls, CSF abnormalities were more common, and two studies found CSF dependent re-diagnosis in 3.2–6%. Current findings suggest that schizophrenia and affective disorders may have CSF abnormalities including signs of blood-brain barrier impairment and inflammation. However, the available evidence does not allow any firm conclusion since all studies showed at least some degree of bias and vastly lacked inclusion of confounding factors. Moreover, only few studies investigated the same parameters with healthy controls and high-quality longitudinal CSF studies are lacking, including impact of psychotropic medications, lifestyle factors and potential benefits of anti-inflammatory treatment in subgroups with CSF inflammation.

## Introduction

Immunological mechanisms in mental disorders have become an area of significant interest [1], and several studies have associated infections and autoimmune diseases with an increased risk of specifically schizophrenia and affective disorders [2–4]. Studies have also shown increased levels of peripheral pro-inflammatory markers [5–11] and genes involved in regulation of the immune system in both schizophrenia and depression [12]. Furthermore, beneficial effects of anti-inflammatory treatment have been found in depression [13] and subgroups with psychotic disorders [14].

However, knowledge is sparse regarding the prevalence of abnormal immunological findings in cerebrospinal fluid (CSF) of people with severe mental disorders. CSF is the biological material closest to the brain that can be easily assessed and lumbar puncture is a routine procedure in neurological but not (yet) in psychiatric practice. Nonetheless, CSF studies have revealed increased CSF/serum albumin ratio in individuals with schizophrenia and affective disorders [6, 15–25] indicating increased blood–brain barrier (BBB) permeability. Other studies found elevated CSF cell count [15, 16, 20, 26, 27], IgG index [16, 21], and the presence of oligoclonal bands [15, 16, 18, 20, 21] which could be indicators of inflammation and intrathecal immunoglobulin production. Also, a meta-analysis found several specific infectious agents to be associated with schizophrenia; however, most studies were based on blood and not CSF, and control groups consisted typically of non-healthy subjects [28]. Another meta-analysis found increased CSF levels of interleukin 1β (IL-1β), IL-6, and IL-8 in patients with severe mental disorders [29]. Despite these intriguing findings, no systematic review has hitherto gathered all the knowledge on infectious and inflammatory CSF abnormalities among patients with severe mental disorders.

Hence, most knowledge on the role of the immune system in mental disorders stems from studies on severe mental disorders, i.e., schizophrenia and affective disorders. Therefore, we aimed to conduct the first systematic review of all CSF studies examining inflammatory markers and infections in persons with schizophrenia spectrum or affective disorders, including meta-analyses of studies with healthy controls. Furthermore, we included the potential clinical implications of a CSF test, risk of adverse events and risk of bias.

## Methods

The study protocol was a priori uploaded on PROSPERO (ID: CRD42017058938) and is available as *online* supplementary file.

### Study selection and search method

In this systematic review we included studies investigating inflammatory markers and infections in the CSF of persons with schizophrenia spectrum disorders or affective disorders, fulfilling the following criteria:

1. Investigation of CSF markers related to inflammation or infections as defined under our primary outcomes.
2. Inclusion of persons diagnosed with schizophrenia spectrum or affective disorders according to Diagnostic and Statistical Manual of Mental Disorders (DSM) or International Classification of Diseases (ICD) or similar classifications that might have been used before DSM and ICD implementation.
3. Publications in peer-reviewed journals.
4. Publications in English.

Studies from all time periods with study subjects in all ages, sexes, and races were included. We included both studies without controls and studies with healthy or non-healthy control groups, but only studies with healthy controls were used for the meta-analysis. The search was performed until March 23, 2017 using PubMed, CENTRAL, EMBASE, Psychinfo, and LILACS with medical subject headings (MESH) or similar when possible or text word terms: (*(psychosis OR psychotic syndrome OR psychotic OR psychotic symptoms OR schizophrenia OR schizophreniform disorder OR schizoaffective OR depression OR major depressive disorder OR bipolar disorder) AND (cerebrospinal fluid OR CSF) AND (blood-brain barrier OR inflammation OR infection OR albumin OR protein OR cell count OR immunoglobulin OR IgG OR oligoclonal bands OR interleukins OR cytokine OR autoimmune disease OR autoimmunity OR immunology OR immune system OR psychoneuroimmunology OR lymphocyte OR macrophage OR C-reactive protein OR autoantibodies OR T cells OR complement))*. Reference lists of relevant reviews were searched for additional studies. Two investigators (SO and SWB) examined titles and abstracts to remove irrelevant reports and examined full texts to determine compliance with inclusion criteria.

### Outcomes

#### Primary outcomes

1. Basic CSF findings: cell count, total protein, albumin, and albumin ratio.
2. CSF inflammatory markers: immunoglobulins, oligoclonal bands, and cytokines.
3. Specific CSF antibodies: antibodies directed against infectious agents as an indicator of preceding or current infection and auto-antibodies.

#### Secondary outcomes

1. Correlation between CSF findings and serum findings, psychotropic medication, and psychiatric symptoms, respectively.
2. Change in diagnosis following CSF analyses.
3. Adverse events in relation to lumbar puncture, e.g., headache.

### Data extraction and bias assessment

Two authors (SO and OKF) extracted data using a pre-piloted structured form not blinded to study results, author names or institutions. This included bibliographical data and participant description. Authors were contacted by e-mail in case of missing details with a reminder sent in case of no response. Two authors (SO and OKF) conducted bias assessment according to the Newcastle-Ottawa criteria for case–control studies as suggested by the Cochrane collaboration (eMethods).

### Statistical analysis

The primary analyses were conducted using RevMan 5.0. When fixed-effects analysis and the random-effects analysis resulted in similar results, only findings from the random effects analysis were reported, but in case of discrepancies both results were reported. We conducted analysis using the standardized mean difference (SMD) since we expected differences in assays. SMD is the mean difference in outcome between cases and controls divided by the pooled standard deviation (SD) giving the result of a unit free effect size. By convention, SMD effect sizes of 0.2, 0.5, and 0.8 are considered small, medium and large effect sizes. Our conclusions were based on the SMD even though we also present results from mean difference. The chi-squared test for heterogeneity will be used to provide an indication of between-trial heterogeneity. In addition, the degree of heterogeneity observed in the results was quantified using the *I*^2^ statistic, which can be interpreted as the percentage of variation observed between the trials attributable to between trial differences rather than sampling error (chance).

### Post-hoc analyses

First, since CSF analysis techniques have improved over time, we performed sensitivity analyses on studies published after the year 2000. Second, we divided patients with psychosis into acute (inpatient treatment) and chronic (recruited in an outpatient setting) psychosis and performed analyses if there were at least two studies in each group, i.e., acute versus chronic.

## Results

### Search results and study characteristics

**Fig. 1:**
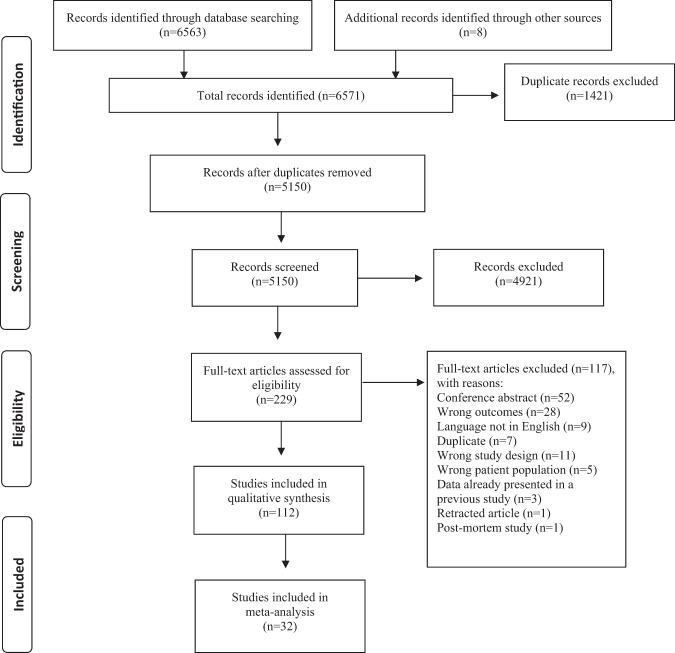
Flowchart of literature search and study selection

**Fig. 2:**
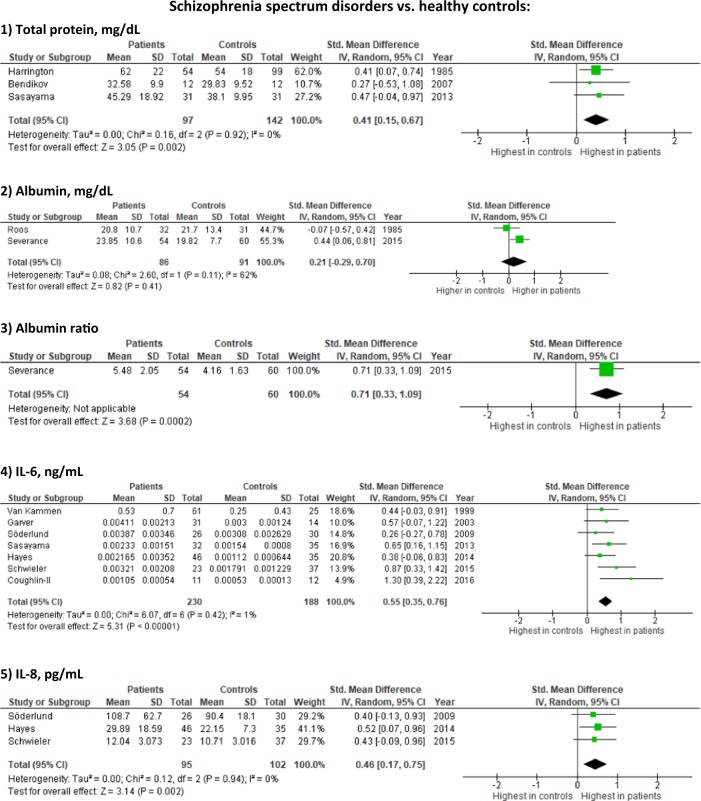

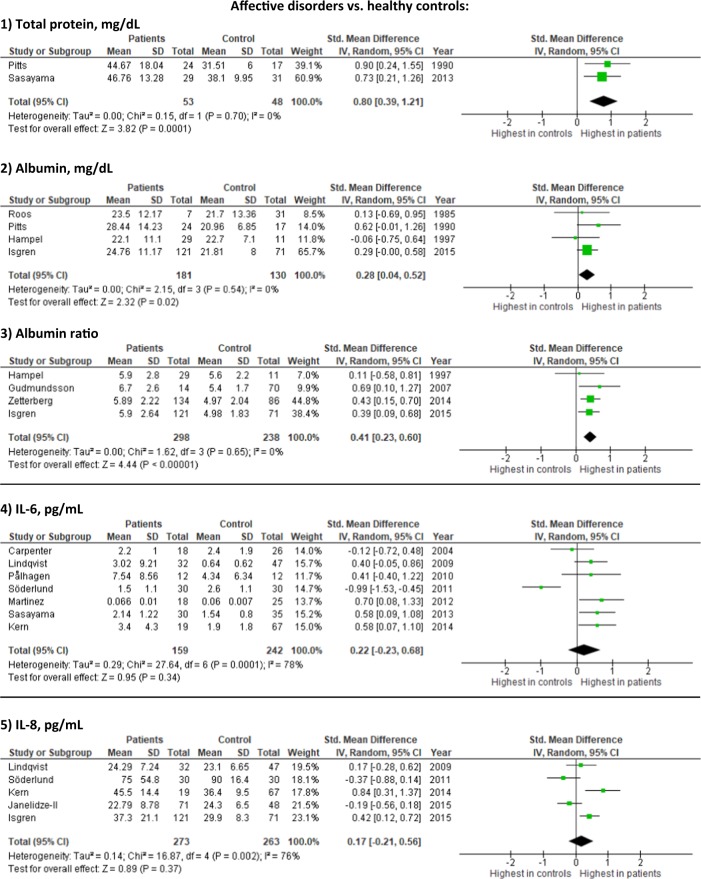
Forest plots on selected results from studies investigating immune-related CSF markers in schizophrenia spectrum and affective disorders (the remaining forest plots are shown in eFigure 1)

We identified 6571 studies (Fig. 1), of which 229 were assessed for full-text inspection. A total of 112 studies investigated CSF immune-related alterations, out of which 38 studies included control groups consisting solely of healthy controls. Data from 32 studies had data necessary for conducting meta-analyses, either published or send to us by the authors. Study characteristics are described in Table 1. Briefly, studies varied regarding gender distribution, age, disease duration, treatment, investigated biomarkers, and whether recruitment happened consecutively or based on clinical indication. In the following sections, the meta-analysis results are presented first (the findings on each specific CSF marker are shown in Table 2 and eTable  1 with forest plots in Figure 2 and eFigure  1) followed by a summary of the remaining CSF studies without healthy controls. Additional analyses only including studies published after the year 2000 supported the primary analyses (eFigure  2). The study characteristics, baseline data, and results for studies without controls or with non-healthy controls are shown in eTables 2 and 3, while results from the studies combining cases with schizophrenia spectrum and affective disorders are presented in the Results.

**Table 1:** Baseline characteristics from CSF studies with healthy control subjects

*Studies included in the meta-analysis*							
Schizophrenia spectrum disorders							
Study	Case subjects			Healthy control subjects		Outcome and results included in meta-analysis	CSF study method
	*N*, diagnosis (diagnostic tool)	No. (%) of males/mean age (year)	Psychotropic medication status	*N*	No. (%) of males/mean age (year)		
Roos [112]^a^	32, schizophrenia (SADS and PSE)	NA/NA	No medication for ≥ 2 weeks	31	NA/NA	Albumin, IgG and IgG-albumin ratio	Isoelectric focusing (IEF)
Harrington [113]	54, schizophrenia (DSM-3)	Male/female ratio = 1.8/35	Two cases without medication	99	Male/female ratio = 1.13/41	Total protein	Two-dimensional electrophoresis
Kirch [25]^b^	46, schizophrenia (DSM-3)	31 (67)/29.9	Some cases without medication ≥ 4 weeks	20	16 (80)/54.1	IgG index. ↑Albumin ratio in 10 cases (22%) and 1 control (5%) *p* = 0.08). ↔ No correlation albumin ratio or IgG with antipsychotic treatment	Patient samples: rate nephelometry Control values: radial immunodiffusion
El-Mallakh [79]	27, schizophrenia, schizoaffect (DSM-3-R)	20 (74)/31.6	Some cases did not receive medication for 4–6 weeks	11	9 (82)/30.3	IL-1alpha and IL-2 ↔ No correlation between IL-2 and PSAS scores	ELISA
Licinio [34]	10, schizophrenia (DSM-3-R)	10 (100)/38.5	No medication ≥ 2 weeks	10	10 (100)/35.1	IL-1alpha and IL-2 cell count normal in cases and controls	ELISA
Katila [35]	14, schizophrenia (DSM-3-R)	9 (64)/42.8	All patients received medication	9	5 (56)/38.3	IL-1beta, normal cell count, IL-6: undetectable in cases and controls ↔ No correlation between serum and CSF IL-1beta	ELISA
Rapaport [114]	60, schizophrenia (DSM-3-R)	34 (57)/29	All patients received medication	21	11 (52)/27	IL-1alpha and IL-2	Competitive enzyme immunoassay
Vawter [115]	44, schizophrenia (NA)	34 (77)/34.5	NA	19	13 (68)/30.2	TGF-beta1, TGF-beta2	ELISA
Van Kammen [82]	61, schizophrenia or schizoaffective disorder (DSM-3-R)	61 (100)/38.1	All patients received medication	25	25 (100)/35.0	IL-6 No correlation between CSF IL-6 and measures of psychosis	ELISA
Nikkilä [44]	19, schizophrenia (DSM-4) for (11 for neopterin, 8 for MIP-1)	Neopterin: 5 (45)/31 MIP-1 alpha: 5 (63)/34	No medication ≥ 3 months	10/8	Neopterin: 7 (70)/34 MIP-1 alpha: 5 (63)/31	Neopterin and MIP-1 alpha. Infection markers, albumin ratio: normal in cases and controls. ↔ No correlation between neopterin or MIP-1 alpha and BPRS symptoms	Radioimmunoassay
Garver [80]	31, schizophrenia (DSM-4)	28 (90)/34.1	No medication for 2–7 days	14	10 (71)/32.9	IL-6 Non-significant trend for IL-6 inversely correlated with SAPS scale	Sandwich enzyme-linked immunosorbent assay
Bendikov [116]	12, schizophrenia (DSM-4)	7 (58)/38.9	10 cases received medication	12	7 (58)/40.5	Total protein	Western blot analysis
Söderlund [90]	26, schizophrenia (DSM-4)	26 (100)/27.5	25 cases received medication	30	30 (100)/25.4	IL-1beta, IL-6, IL-8. IL-2, IL-4, GM-CSF, IFN-gamma, TNF-alpha, IL-5, IL-10: not detectable or only in low concentration. ↔ No correlation IL-1β, IL-6 or IL-8 and antipsychotic dose	Sandwich-immunoassay-based protein-array system
Sasayama [84]^a^	32, schizophrenia (DSM-4)	20 (63)/40.8	Most cases received medication	35	21 (60)/41.3	IL-6, cell count and total protein. ↔ No correlation between serum and CSF IL-6 and antipsychotic dose or PANSS symptoms	ELISA
Hayes [117]	46, schizophrenia (DSM-4)	36 (78)/25.8	No medication	35	21 (60)/26.4	IL-6, IL-6R, IL-8, C3, MCP-2, and TNFR2	ELISA
Schwieler [85]	23, schizophrenia and schizoaffective disorder (DSM-4)	15 (65)/median men = 37, women = 35	All cases received medication	37	23 (62)/men: median 24; women: median 23	IL-6, IL-8. IFN-gamma, IL-1beta, IL-4: undetectable IL-2, IL-10, IL-18, TNF-alpha, IFN-alpha-2a: only detected in limited number of CSF samples ↔ No correlation IL-6 or IL-8 and symptoms (GAF, BPRS)	Electrochemiluminescence detection Liquid chromatography/mass spectrometry
Severance [118]^c^	105, schizophrenia (DSM-4)	67 (64)/28.62	75 cases without medication	61	30 (49)/27.16	Albumin, albumin ratio, IgG, IgG ratio, IgG index	ELISA
Coughlin [102]^d^	14, schizophrenia (DSM-4)	11 (79)/24.1	2 cases without treatment for 1 month	16	9 (56)/24.9	IL-6. Correlation between CSF and plasma IL-6 within the total study population and the patient cohort alone	V-Plex Custom Human Biomarkers kit
*Affective disorders*							
Roos [112]^a^	7, depression (SADS and PSE)	NA	No medication ≥ 2 weeks	31	NA	Albumin, IgG and IgG-albumin ratio	Isoelectric focusing (IEF)
Pitts [46]^e^	24, Affective disorders (DSM-3-R)	13 (54)/37.5	Most patients received medication	17	8 (47)/29.8	Total protein and albumin. Normal cell count in cases and controls ↔ No correlation total protein and depressive symptoms (HAMD)	Protein electrophoresis
Hampel [119]	29, MDD (DSM-3-R and ICD-10)	9 (31)/71.6	NA	11	6 (55)/72.8	Albumin, albumin ratio, IgG, and IgG ratio	Immunonephelometric
Hampel [120]^f^	29, MDD (DSM-3-R and ICD-10)	9 (31)/71.6	NA	11	6 (55)/72.8	IgG index and oligoclonal bands	Isoelectric focusing (IEF)
Carpenter [121]	18, unipolar depression (DSM-4)	9 (50)/38.3	No major psychotropic drugs for ≥ 3 weeks	26	12 (46)/32.7	IL-6	ELISA
Gudmundsson [89]	14, MDD and dysthymia (DSM-3-R)	0 (0)/NA	NA	70	0 (0)/NA	Albumin ratio ↔ No correlation between biomarker levels and psychotropic medication	ELISA
Lindqvist [8]^g^	32, MDD or depression NOS (DSM-3)	NA/NA	No medication for 16 days (mean)	47	40 (85)/37	IL-1 beta, IL-6, IL-8, and TNF-alpha	ELISA
Pålhagen [122]	12, depression (DSM-3/4)	7 (58)/62.7	No antidepressants	12	12(100)/29.4	IL-6	Mass fragmentographic method
Söderlund [123]	30, bipolar disorder type 1 or 2 (DSM-4)	30 (100)/43.2	2 cases without medication	30	30 (100)/25.4	IL-1 beta, IL-6, and IL-8. IL-2, IL-4, IL-5, IL-10, GM-CSF, IFN-γ, TNF–α: undetectable or close to the detection limit of the assay	Immunoassay-based protein array multiplex system
Martinez [124]	18, MDD (DSM-3-R)	8 (44)/40.4	No medication for 2 weeks	25	13 (52)/29.9	IL-1, IL-6 and TNF-alpha	ELISA
Janelidze [81]^g^	75, MDD, dysthymia, depression NOS or bipolar (DSM-3-R)	NA/NA	No medication for 14.7 days (mean)	43	36 (84)/38.8	IP-10, Eotaxin, MIP-1b, MCP-1, MCP-4 and TARC	Ultra-sensitive chemokine multiplex immunoassay
Sasayama [84]^a^	30, MDD (DSM-4)	19 (63)/42.7	Most subjects received medication	35	21 (60)/41.3	IL-6, cell count and protein. ↔ No correlation CSF and serum IL-6, IL-6 and antidepressant dose or HAMD-21 symptoms	ELISA
Kern [77]^h^	19, Major and minor depression (DSM-4)	0 (0)/72.8	5 cases received antidepressant medication	67	0 (0)/72.4	IL-6, IL-8. No correlation CSF IL-6 and MADRS symptoms (*p* = 0.450) Higher CSF IL-8 correlated with higher MADRS scores (*p* = 0.003)	Human Pro-inflammatory II 4-Plex Assay Ultra- Sensitive Kit
Zetterberg [88]	134, bipolar, schizoaffective manic type, cyclothymia (DSM-4)	54 (40)/36 (28–50) = median (IQR)	NA	86	38 (44)/34 (28–46) = median (IQR)	Albumin ratio. ↔ No correlation between albumin ratio and psychotropic medication, apart from antipsychotic medication. Higher albumin ratio in those treated with antipsychotic medication compared to those treated with other psychotropic medication	Immunonephelometry
Isgren [86]	121, bipolar spectrum disorder (DSM-4-TR)	47 (38.8)/36.0	Most cases received medication	71	26 (36.6)/32.0	Albumin ratio, albumin, and IL-8. Correlation IL-8 with lithium and antipsychotic. ↔ No correlation IL-8 and other drugs or psychiatric symptoms (CGI, MADRS, YMRS)	Immunoassays
Janelidze [87]^g^	71, MDD, depr NOS, dysthymia (DSM-3-R)	NA/NA	No medication for 15 days (mean)	48	39 (81)/38	IL-8. ↔ No correlation IL-8 and MADRS symptoms (*p* > 0.05)	ELISA
*Studies not included in the meta-analysis*							
Ueno [125]^i^	139, schizophrenia (NA)	83 (60)/Range 12–67	NA	6	NA	Lower total protein in cases, no information regarding statistical difference to controls	Polarographic method and paper electrophoresis
Deutsch [43]	19, schizophrenia, 11, depression (NA)	NA/33.6, 48.1 and 63.8	NA	6	NA/30.5	↔ Total protein: no difference between cases and controls after adjustment for age (*p* > 0.10)	Liquid scintillation spectrometry
Preble [126]	65, MDD, schizophrenia spectrum, bipolar, schizotypal personality disorder, (DSM-3)	NA	NA	20	NA	Interferon: not present in cases or controls CMV IgM: present in 9 cases (13.8%)	ELISA
Pazzaglia [27]^j^	240, bipolar disorder or unipolar recurrent depression (DSM-3)	95 (40)/ NA	No medication ≥ 2 weeks	55	34 (62)/ NA	↑Cell count in 3 cases (1.25%), normal in controls. ↑Total protein in cases compared with controls. ↔ No correlation total protein and psychiatric symptoms (Bunney-Hamburg Rating Scale)	DuPont ACA discrete clinical analyzer
Stübner [62]	20, MDD (DSM-3-R and ICD-10)	7 (35)/67.3	1 case without medication	20	12 (60)/65.8	↓IL-6 and sIL-6r in cases compared with controls (*p* < 0.001) ↔ sgp130: no difference between cases and controls (*p* = 0.152)	ELISA
Leweke [36]	85, schizophrenia anti-psychotic naïve: 36^k^, first psychotic episode; 10 prior antipsychotic treatment; 39 inpatients medicated (DSM-4)	28 (78), 8 (80) and 30 (77)/29.1, 33.7 and 30.5	According to group	73	52 (71)/28.0	Cell count, albumin ratio, oligoclonal bands, IgG index: normal in cases ↑CMV (*p* < 0.0033) and toxoplasma gondii (*p* < 0.001) IgG in cases from group 1 compared with controls ↔ CMV and toxoplasma IgG for group 2 + 3 and HSV-1 IgG for all groups did not differ from controls	ELISA
Coughlin [127]	15, schizophrenia (NA)	11 (73)/21.9	1 case without medication	15	12 (80)/23.6		ELISA

*NA* not available, *MDD* major depressive disorder, *DSM* diagnostics and statistics manual, *ICD* International Classification of Diseases, *PSE* present stata examination, *SADS* schedule for affective disorders and schizophrenia, *HAMD* Hamilton depression score, *MADRS* Montgomery and Aasberg depression rating scale, *YMRS* young mania rating scale, *CGI*, clinical global impression scale, *PSAS* psychiatric symptom assessment scale, *BPRS* brief psychiatric rating scale, *PANSS* positive and negative symptom scale, *SAPS* scale for assessment of positive symptoms, *GAF* global assessment of functioning, *IL* interleukin, *IFN* interferon, *TNF* tumor necrosis factor, *Ig* immunoglobulin, *TGF* transforming growth factor, *GM-CSF* granulocyte-macrophage colony-stimulating factor, *MIP* macrophage inflammatory protein, *MCP* monocyte chemoattractant protein, *IP* inducible protein, *CMV* cytomegalovirus, *HSV* Herpes simplex virus

^a^The study included both cases with psychosis and affective disorders and is thus shown in the table twice

^b^Data on 24 of the 46 cases is presented in Kirch et al. [21], but that study is not included in the meta-analysis

^c^Some analyses were performed on a smaller subsample

^d^Only 11 cases and 12 controls underwent LP

^e^Only 13 patients and 8 controls were included in the albumin analyses

^f^Same patients as in Hampel [119], only new results included in the meta-analysis

^g^Patients were examined after a suicide attempt

^h^The cases were included from the same original cohort used in Gudmundsson [89], however with the investigation of other CSF outcomes

^i^Some analyses were only performed in subgroups of the cases

^j^125 patients and 8 controls had >1 medication-free LP and in these cases, mean values were used for most analyses

^k^2 cases from group 1 did not have available CSF samples

**Table 2:** CSF immune related markers in patients with schizophrenia spectrum or affective disorders compared to healthy controls

CSF Marker	Studies	Cases	Control	SMD	95% CI	*p*-value	*I* ^2^
*Schizophrenia vs healthy controls*							
Cell count	1	32	31	0.19	−0.31 to 0.68	0.46	NA
Total protein	3	97	142	**0.41**	0.15 to 0.67	**0.002**	0%
Albumin	2	86	91	0.21	−0.29 to 0.70	0.41	62%
Albumin ratio	1	54	60	**0.71**	0.33 to 1.09	**0.0002**	NA
IgG	2	86	91	−0.12	−0.93 to 0.69	0.77	85%
IgG Ratio	1	54	60	**0.68**	0.30 to 1.06	**0.0004**	NA
IgG/Albumin ratio	1	32	31	**−0.62**	−1.13 to −0.12	**0.02**	NA
IgG Index	2	100	80	0.25	−0.07 to 0.56	0.13	7%
IL-1 alpha	3	72	33	−0.16	−0.91 to 0.60	0.68	43%
IL-1 Beta	2	40	39	−0.13	−4.96 to 4.71	0.96	98%
IL-2	3	97	42	0.18	−0.49 to 0.85	0.60	64%
IL-6	7	230	188	**0.55**	0.35 to 0.76	**<0.00001**	1%
IL-6R	1	46	35	−0.24	−0.68 to 0.20	0.28	NA
IL-8	3	95	102	**0.46**	0.17 to 0.75	**0.002**	0%
Neopterin	1	11	10	−0.05	−0.91 to 0.81	0.91	NA
MIP-1 alfa	1	8	8	−0.70	−1.72 to 0.32	0.18	NA
C3	1	46	35	0.00	−0.44 to 0.44	1.00	NA
MCP-2	1	46	35	0.26	−0.18 to 0.71	0.24	NA
TNFR2	1	46	35	0.06	−0.38 to 0.50	0.78	NA
TGFB1	1	44	19	0.29	−0.25 to 0.83	0.29	NA
TGFB2	1	44	19	−0.14	−0.68 to 0.40	0.61	NA
*Affective Disorders vs healthy controls*							
CSF Marker	Studies	Cases	Control	Mean ES	95% CI	*p*-value	*I* ^**2**^
Cell count	1	29	31	0.40	−0.11 to 0.91	0.13	NA
Total protein	2	53	48	**0.80**	0.39 to 1.21	**0.0001**	0%
Albumin	4	181	130	**0.28**	0.04 to 0.52	**0.02**	0%
Albumin ratio	4	298	238	**0.41**	0.23 to 0.60	**<0.00001**	0%
IgG	2	36	42	−0.22	−0.75 to 0.32	0.43	0%
IgG Ratio	1	29	11	0.33	−0.37 to 1.02	0.36	NA
IgG/Albumin ratio	1	7	31	−0.56	−1.39 to 0.28	0.19	NA
IgG Index	1	29	11	0.22	−0.48 to 0.91	0.54	NA
IL-1	1	18	25	0.61	−0.01 to 1.23	0.05	NA
IL-1 Beta	2	62	77	0.80	−0.99 to 2.59	0.38	96%
IL-6	7	159	242	0.22	−0.23 to 0.68	0.34	78%
IL-8	5	273	263	0.17	−0.21 to 0.56	0.37	76%
TNF-alpha	2	50	72	0.24	−0.12 to 0.61	0.19	0%
Eotaxin-1	1	75	43	−0.33	−0.71 to 0.04	0.08	NA
IP-10	1	75	43	−0.17	−0.55 to 0.20	0.37	NA
MIP-1B	1	75	43	−0.26	−0.64 to 0.12	0.17	NA
MCP-1	1	75	43	−0.28	−0.65 to 0.10	0.15	NA
MCP-4	1	75	43	**−0.76**	−1.15 to −0.37	**0.0001**	NA
TARC	1	75	43	**−0.57**	−0.95 to −0.19	**0.004**	NA

### Bias assessment

All studies included in the meta-analysis showed at least some degree of bias (eTable 4). The majority of studies were biased concerning the actual case definition (16/32), representativeness of cases (28/32), random selection of controls (20/32), comparability between cases and controls (21/32) and ascertainment of exposure (27/32).

## Primary outcomes

### CSF cell count, total protein, albumin, and albumin ratio

#### Schizophrenia spectrum disorders

In the meta-analysis comparing to healthy controls, total protein (3 studies [97 patients]; SMD: 0.41; 95% CI 0.15–0.67; *I*^2^ = 0%) and albumin ratios (1 study [54 patients]; SMD: 0.71; 95% CI 0.33–1.09) were elevated, whereas albumin and cell counts were not significantly increased.

Across all CSF studies, cell counts ranged from normal [18, 19, 23, 30–36] to increased levels in up to 3.4% of the cases [20, 26] with no difference comparing to neurological controls [37]. Total protein levels varied from normal [18, 22, 30, 31] to increased in up to 42.2% of cases [6, 20, 26, 38, 39], with no difference in the studies comparing to neurological, psychiatric, surgical or healthy controls [32, 40–43]. Albumin levels ranged from normal [18, 22, 30, 31] to increased in up to 16% of cases [6, 24, 39], with no difference in the studies comparing with psychiatric controls [40] or a combined group of healthy and psychiatric controls [38]. The albumin ratio ranged from normal [36, 37, 44, 45] to increased in up to 53% of the cases [6, 19–25], with no difference in the studies comparing to healthy controls [25] or a combined group of healthy and psychiatric controls [38].

#### Affective disorders

In the meta-analysis comparing to healthy controls, cell count was not significantly increased, whereas total protein levels (2 studies [53 patients]; SMD: 0.80; 95% CI 0.39–1.21, *I*^2^ = 0%), albumin (4 studies [181 patients]; SMD = 0.28; 95% CI 0.04–0.52, *I*^2^ = 0) and albumin ratio were increased (4 studies [298 patients]; SMD = 0.41; 95% CI 0.23–0.60, *I*^2^ = 0).

Across all CSF studies, cases had normal [18, 46] or increased cell counts in up to 13.1% [15, 16, 27], with no difference in the studies comparing to neurological controls [15, 47]. Total protein ranged from normal [18, 47, 48] to increased levels in up to 36.6% of cases [16, 27, 49], with no difference in the studies comparing to psychiatric or neurological controls [16, 40, 47]. Albumin was not different compared to somatically ill or psychiatric controls [40, 50]. The albumin ratio was increased in up to 44% of cases [15–18] but with no difference in the studies comparing with neurological controls [15, 16].

### CSF immunoglobulins

#### Schizophrenia spectrum disorders

In the meta-analysis comparing to healthy controls, IgG/albumin ratio was decreased (1 study [32 patients]; SMD = −0.62; 95% CI −1.13 to −0.12), IgG ratio was increased (1 study [54 patients]; SMD = 0.68; 95% CI 0.30–1.06), whereas IgG levels and the IgG index were not significantly increased.

In the other CSF studies, immunoglobulins were increased in 3% of the cases [51], whereas IgG and the IgG index were normal [19, 22, 36, 50] or increased in up to 33% [6, 19, 21, 24, 52] of cases, respectively. In the studies comparing with surgical, neurological or a combined group of psychiatric and healthy controls, the IgG ratio, IgG, IgA and IgM were decreased [32, 53] or not different in cases [22, 38, 50]. Oligoclonal bands were absent in some reports [18, 36, 54] but present in up to 12.5% of cases in others [20, 21]. An intrathecal immunoglobulin synthesis was observed in none [18] to 7.2% of cases [20].

#### Affective disorders

In the meta-analysis, IgG levels, the IgG/albumin ratio, the IgG ratio and the IgG index were not significantly different compared to healthy controls.

In studies not included in the meta-analysis IgG was present in all cases [55], with increased levels of IgG [49] but within normal range IgG ratio [16]; however, the IgG index was significantly higher in cases in the one study comparing with neurological controls [16]. IgM was not present in cases [49, 55] while IgA was present in 23.3% [49, 55]. Oligoclonal bands were found in up to 12.5% of cases [15, 16, 18] with no difference in the studies comparing to neurological controls [15, 16]. Intrathecal immune response was present in none [18] to 30% of cases and significantly increased in the study comparing with neurological controls [16].

### CSF interleukins

#### Schizophrenia spectrum disorders

In the meta-analysis comparing to healthy controls, IL-8 (3 studies [95 patients]; SMD = 0.46; 95% CI 0.17–0.75; *I*^2^ = 0%) and IL-6 (7 studies [230 patients]; SMD = 0.55; 95% CI 0.35–0.76; *I*^2^ = 1%) were significantly increased. In a post-hoc analysis, we found that IL-6 was significantly elevated in acute psychosis (SMD = 0.46; 95% CI 0.22–0.71; *I*^2^ = 1%) and chronic psychosis (SMD = 0.75; 95% CI 0.39 to 1.12; *I*^2^ = 0%) with the between-group difference being not significant (*p* = 0.20) (eFigure 3). The levels of IL-1alpha, IL-1beta and IL-2 were not statistically different from healthy controls.

In the studies not included in the meta-analysis, the anti-inflammatory cytokines IL-4 and IL-10 were present in 64 and 14% of cases, respectively. Concerning pro-inflammatory cytokines, IL-2 was detected in 95%, IL-5 in 40%, IFN-gamma in 14%, TNF-beta in 41%, and TNF-alpha in 50% of cases [7]. Other studies found no difference to neurological controls in levels of IL-2, IL-6, and TNF alpha [33] or decreased levels of IL-1beta, sIL-2r [33], and TNF-alpha [56] compared to neurological and surgical controls. Interferon was reported to be absent [31] or present in up to 59% of cases [57–60] but with no difference in the study comparing to psychiatric controls [60].

#### Affective disorders

In the meta-analysis comparing to healthy controls, IL-1, IL-1beta, IL-6, IL-8, and TNF-alpha were not significantly increased (Table 2, Fig. 2 and eFigure 1).

The studies comparing neurological [47, 61] and healthy [62] controls that were not included in the meta-analysis, showed that cases had decreased or unchanged levels of IL-6 [61–63], decreased levels of sIL-6r [62] and sIL-2r [47], increased levels of IL-1beta [61, 63], and unchanged levels of TNF-alpha [61] and sgp130 [62]. Moreover, a wide range of inflammatory markers were found to be similar in psychiatric patients compared to neurological controls (eTable 1) [63]. IL-7, IL-12, or granulocyte-colony stimulating factor (G-CSF) were not detected [63].

### Specific CSF antibodies

#### Schizophrenia spectrum disorders

One study with healthy controls found increased CMV and toxoplasma gondii IgG in antipsychotic naïve cases [36], but the data were not suitable for meta-analysis. Other studies found that CMV antibodies [48, 64–68] were either undetectable [48, 64–68] or present (in up to 18.5% of cases; 43,66,67), but this was not different from surgical [32] or psychiatric and healthy controls [38, 69]. HSV-1 or 2 antibodies were undetectable in one [19] but detectable in the other studies (present in up to 69% of cases) [48, 57–59, 64, 67, 70], but without difference in the studies comparing to neurological, surgical controls, psychiatric or healthy controls [30, 32, 36, 38].

Studies found antibodies in cases against mumps in 2.9% [64] (but decreased compared with surgical controls [32]), VZV in 5.7% [64], tick-borne encephalitis virus in 7.3%, orbivirus lipovnik in up to 2.9%, choriomeningitis virus in 5.3% [57], and nucleotide sequences homologous to those of known retroviruses in 20% of cases [71], whereas others did not find CSF antibodies against infectious agents [19, 30, 39, 64, 66, 72, 73]. There was no difference in the levels of antibodies towards measles [32], rubella [30, 32], VZV [32], adenovirus [32], vaccinia [38], or influenza [38] between cases and surgical [30, 32], psychiatric or healthy controls [38].

Antibodies against neuronal cell surface antigens were detected in up to 2.4% and against intracellular onconeural antigens in up to 2.1% of cases but none against intracellular synaptic antigens in cases [20]. Others found anti-brain antibodies in 48.1% of cases [74], with a study finding dopamine IgG in 100% of cases and significantly elevated compared to the 41% of neurological controls [22]. No difference of antibodies against myelin basic protein and glial fibrillary acidic protein was observed compared to surgical controls [31].

#### Affective disorders

Studies found CSF antibodies against toxoplasma gondii in 52.5% [16], HSV in up to 85%, EBV in up to 60% [16, 48], CMV in 3% [72] and BDV in up to 50% [16, 45] of cases. Others found no antibodies against measles [48], CMV [48], or treponema pallidum [73] in cases. There were no antibodies against intracellular antigens or neuronal cell surface antigens [75, 76].

## Secondary outcomes

### Correlation between CSF findings and psychiatric symptoms

Correlations have been found between albumin and IgG with SANS scores [6], and higher IL-8 levels with higher MADRS scores [77]. However, most authors reported no correlation between psychiatric symptoms and the following CSF findings: total protein [27, 46], impairment of the BBB [51], IL-1alpha [78], IL-1beta [61], IL-2 [78, 79], IL-6 [80–85], sIL-6r [24], IL-8 [85–87], IL-10 [83], IL-15 [63], TNF-alpha [56], neopterin [44], MIP-1alpha [44], MCP-1 [63], and lymphocyte activational stage [40].

### Correlation between CSF findings and psychotropic medication

A total of 17 studies investigated the association between medication and CSF findings. CSF analysis in patients on antipsychotic medication revealed a tendency towards normalization of the CSF cytological alterations [37]. There was a correlation between higher albumin ratio and IL-1alpha with antipsychotic treatment [78, 88], IL-8 and the use of lithium or antipsychotics but not the use of other psychotropic medication [86]. However, most studies found no correlation between antipsychotic or antidepressant medication and CSF levels of IgG, IgM, IgA [53], IgG index [25], total protein [73], albumin ratio [88, 89], CMV IgM [45], lymphocytic profile [23], IL-1 beta, IL-2, sIL-2r, IL-6, IL-8, and TNF alpha [33, 78, 84, 85, 90], or impairment of the BBB [19, 21, 25, 51, 91].

### Change of diagnosis after CSF analyses

Two studies reported that 3.2% (*N* = 5/155) [92], respectively, 6% (*N* = 4/63) [93] of patients with initial diagnoses of affective [93] or schizophrenia spectrum disorders [92, 93] received a revised diagnosis following CSF analyses, including infections and autoimmune disorders.

### Adverse events after lumbar puncture

Only one study reported on adverse events related to the lumbar puncture and found that mild to moderate adverse events (mostly headaches or local pain at the puncture site) occurred in 10.3% of the cases; 1.3% experienced severe post-lumbar puncture headache with nausea [92].

## Discussion

This is the first systematic review and meta-analysis of the available evidence from eight decades on immune-related CSF investigations in schizophrenia and affective disorders, including previously unpublished data acquired from contacts to the study authors. Our meta-analysis pointed towards BBB impairment with increased albumin ratio and total protein in schizophrenia and affective disorders, and increased levels of albumin in affective disorders. The increased IgG CSF/serum ratio (and a non-significantly increased IgG index) in schizophrenia might suggest intrathecal IgG production. Moreover, IL-6 and IL-8 levels were increased in schizophrenia but not significantly increased in affective disorders. However, all studies included in the meta-analysis showed at least some degree of bias, specifically concerning representativeness of cases and ascertainment of exposure.

The major limitation was the small number of studies with healthy controls; hence, the largest meta-analysis included 4 studies with 302 cases. Several meta-analyses were based on one study. The remaining part of the systematic review consisted of studies with non-healthy control groups or without a control group lowering the reliability. Second, detailed study protocols were commonly unavailable, case groups were often unsystematically identified and consisted of various diagnostic categories with variable disease duration, severity and age of onset, making it difficult to apply the results to specific patient groups. Third, CSF analysis varied according to sampling time points and sensitivity of the cytokine assays, and many studies did not disclose the number of samples above the detection limit. In addition, assays have developed over time and hence studies used different techniques, and particularly the broad spectrum of new and more sensitive methods has been emphasized by several studies [94, 95]. Fourth, the majority of studies were cross-sectional. Fifth, we could not perform meta-analyses on studies investigating the importance of psychotropic medication nor could we identify longitudinal studies investigating psychotropic medication. Furthermore, studies with healthy controls examining a broad range of immune-related CSF markers were lacking. Sixth, we only included articles written in English and geographic biases might occur as clinicians may be more likely to perform lumbar punctures in areas where infections with treatable infectious agents are prevalent. Seventh, several studies did not match on important factors such as age or gender (eTable 4) and did not control for important confounders such as BMI, smoking or diet. These confounding factors have been shown to largely influence the associations between at least peripheral inflammatory markers and depression [96], which need to be considered in future studies also for CSF inflammatory markers. Finally, we had no knowledge regarding longitudinal data relating to clinical state and severity of the disorder or response to medications.

The major findings in our meta-analysis were increased albumin ratios and total protein suggestive of BBB leakage or dysfunction. Supporting this, studies have found an increased albumin ratio in up to 53% with schizophrenia spectrum disorders [6, 19–25] and 44% with affective disorders [15–18]. An impaired BBB may leave the brain more vulnerable to harmful substances in the blood, including immune components. It might also be an indicator of inflammation within the central nervous system (CNS), which is also indicated by the non-significantly increased CSF cell counts.

Our meta-analysis reveals evidence for intrathecal IgG production in a subset of psychiatric patients [20, 93] and oligoclonal bands were increased in up to 12.5% of cases [15, 16, 18, 20, 21], which may indicate acute or chronic inflammation, an “immunological scar” from previous inflammation of brain tissue, immunoglobulin production or a local B cell immune response in certain subgroups of patients [97].

Interleukins are produced by the immune system and regulate many aspects of inflammation and the immune response. The present meta-analyses showed increased levels of IL-6 and IL-8 in schizophrenia spectrum disorders, whereas in affective disorders all cytokines levels were non-significantly increased. Other recent meta-analyses revealed evidence of increased CSF levels of IL-1β in schizophrenia and bipolar disorders and increased levels of IL-6 and IL-8 in schizophrenia and depression [29, 98]. IL-6 stimulates CRP production by hepatocytes, and IL-8 primarily induces chemotaxis and phagocytosis. Increased levels of several peripheral cytokines have also been reported for schizophrenia, bipolar disorder and depression [99–101]. Although CSF cytokine levels reflect CNS inflammation more precisely, peripheral IL-6 can reach the CNS through the choroid plexus or because of increased BBB permeability [102].

The studies on CSF antibodies could not be included in meta-analyses. Nonetheless, studies found antibodies against CNS tissue in up to 100% [20, 22, 74] and HSV-1 antibodies in up to 69% of cases with schizophrenia spectrum disorders [48, 57–59, 64, 70], but the results for most of the other infectious agents were rather conflicting. Antibodies against HSV [16, 48], Toxoplasma gondii [16] and EBV [16, 48] had the strongest associations with affective disorders. Furthermore, studies without healthy controls have found signs of CNS pathology in up to 41% of cases [93].

Antipsychotic medication has been found to increase BBB permeability in animal studies [103] and affect immune cells in the CNS [23, 104–106], highlighting the importance of evaluating the effect of psychotropic medication when analyzing CSF; however, the evidence for antidepressants is conflicting [107, 108]. Cross-sectional studies mostly found no association between CSF parameters and psychotropic medication apart from a correlation between a higher albumin ratio [88], higher IL-1alpha [78], and IL-8 [86] with antipsychotic treatment, and none of the studies investigating this aspect longitudinally.

Concerning associations with clinical symptomatology, the only CSF parameters that correlated with symptom scores were IL-8 (depression) [77], respectively albumin and IgG (schizophrenia) [6]. Increased inflammatory blood markers (e.g., CRP and IL-6) correlated with greater overall symptom severity in patients with depression, in particular with neuro-vegetative symptoms (e.g., sleep, appetite) [109–111]. However, only few studies explored associations between CSF markers and symptom severity or correlations between immune markers in serum vs. CSF, and we found conflicting results regarding correlation between IL-6 levels in CSF and serum [84, 102].

### Conclusion and perspectives

The present systematic review and meta-analysis suggests that subgroups of patients with schizophrenia spectrum or affective disorders may have CSF pathology with signs of BBB impairment, intrathecal antibody synthesis and elevated levels of inflammatory markers, autoantibodies and immunoglobulins. However, CSF findings varied greatly and important confounders were often not accounted for limiting any firm conclusions regarding CSF pathology in patients with depression or schizophrenia. Therefore, future studies should be longitudinal with systematic and standardized collection of CSF samples over time in larger study populations with healthy control subjects. Preferably, these studies should include newly diagnosed patients who are naive to psychotropic drugs, with CSF measurements prior to and at several time points after psychotropic drug initiation, with adjustments for variables that can affect the immune-related markers, e.g., smoking and BMI. CSF and peripheral immune markers, in combination with peripheral blood tests and brain scans, might aid in future trials on immune-modulating add-on treatment for subgroups of mental disorders. Finally, the emerging role of the immune system and CNS inflammation in mental disorders necessitates improved imaging methods, better methods for sampling small amounts of CSF using small needles and guided insertions and identification of brain derived proteins in blood. Adverse events after lumbar puncture was rare and 3.2–6% of patients received a revised (somatic) diagnosis following CSF analysis, suggesting that lumbar puncture can be an important supplemental diagnostic examination in psychiatric patient’s potentially influencing treatment.

## Electronic supplementary material

Supplemental material
