# Correction: Cerebrospinal fluid markers of inflammation and infections in schizophrenia and affective disorders: a systematic review and meta-analysis

**DOI:** 10.1038/s41380-019-0381-9

**Published:** 2019-03-12

**Authors:** Sonja Orlovska-Waast, Ole Köhler-Forsberg, Sophie Wiben Brix, Merete Nordentoft, Daniel Kondziella, Jesper Krogh, Michael Eriksen Benros

**Affiliations:** 1 Mental Health Centre Copenhagen, University of Copenhagen, Faculty of Health Sciences, Copenhagen, Denmark; 2 iPSYCH The Lundbeck Foundation Initiative for Integrative Psychiatric Research, Aarhus, Denmark; 3 Psychosis Research Unit, Aarhus University Hospital, Risskov, Denmark; 4 Department of Clinical Medicine, Aarhus University, Aarhus, Denmark; 5 Department of Neurology, Rigshospitalet, Copenhagen University Hospital, Copenhagen, Denmark; 6 Department of Neuroscience, Norwegian University of Science and Technology, Trondheim, Norway

**Keywords:** Diagnostic markers, Schizophrenia, Depression

**Correction to:** **Molecular Psychiatry**;

10.1038/s41380-018-0220-4;

published online 16 August 2018

Following publication of this article, the authors discovered errors in their reporting of the results on IL-6. These mistakes have now been corrected in the published article. The changes made to the original article are detailed below:

1) In the ‘Abstract’:

“The CSF/serum albumin ratio was increased in schizophrenia (1 study [54 patients]; SMD = **0.62**; 95% CI **0.24–1.00**) and affective disorders (4 studies [**302** patients]; SMD = **0.43**; 95% CI **0.25–0.61**, I^2^ = 0%), compared to healthy controls. Total CSF protein was elevated in both schizophrenia (3 studies [97 patients]; SMD = **0.38**; 95% CI **0.12–0.65**, I^2^ = 0%) and affective disorders (2 studies [53 patients]; SMD = **0.77**; 95% CI **0.36–1.18**, I^2^ = 0%). The IgG ratio was increased in schizophrenia (1 study [54 patients]; SMD = **0.60**; 95% CI **0.23–0.98**), whereas the IgG Albumin ratio was decreased (1 study [32 patients]; SMD = −0.62; 95% CI −1.13 to −0.12). Interleukin-6 (IL-6) levels (7 studies [230 patients]; SMD = **0.38**; 95% CI **0.02–0.74**; I^2^ = **64%**) and IL-8 levels (3 studies [95 patients]; SMD = 0.46; 95% CI 0.17–0.75, I^2^ = 0%) were increased in schizophrenia but not significantly increased in affective disorders.”

was changed to:

“The CSF/serum albumin ratio was increased in schizophrenia (1 study [54 patients]; SMD = **0.71**; 95% CI **0.33-1.09**) and affective disorders (4 studies [**298** patients]; SMD = **0.41**; 95% CI **0.23–0.60**, *I*^2^ = 0%), compared to healthy controls. Total CSF protein was elevated in both schizophrenia (3 studies [97 patients]; SMD = **0.41**; 95% CI **0.15–0.67**, *I*^2^ = 0%) and affective disorders (2 studies [53 patients]; SMD = **0.80**; 95% CI **0.39–1.21**, *I*^2^ = 0%). The IgG ratio was increased in schizophrenia (1 study [54 patients]; SMD = **0.68**; 95% CI **0.30–1.06**), whereas the IgG Albumin ratio was decreased (1 study [32 patients]; SMD = −0.62; 95% CI −1.13 to −0.12). Interleukin-6 (IL-6) levels (7 studies [230 patients]; SMD = **0.55**; 95% CI **0.35–0.76**; *I*^2^ = **1**%) and IL-8 levels (3 studies [95 patients]; SMD = 0.46; 95% CI 0.17–0.75, *I*^2^ = 0%) were increased in schizophrenia but not significantly increased in affective disorders.”

2) In the section ‘CSF cell count, total protein, albumin, and albumin ratio’ under the heading ‘Schizophrenia spectrum disorders’:

“In the meta-analysis comparing to healthy controls, total protein (3 studies [97 patients]; SMD: 0.41; 95% CI 0.15–**0.68**; I^2^ = 0%) and albumin ratios (1 study [54 patients]; SMD: **0.62**; 95% CI **0.24–1.00**) were elevated, whereas albumin and cell counts were not significantly increased.”

was changed to:

“In the meta-analysis comparing to healthy controls, total protein (3 studies [97 patients]; SMD: 0.41; 95% CI 0.15–**0.67**; *I*^2^ = 0%) and albumin ratios (1 study [54 patients]; SMD: **0.71**; 95% CI **0.33–1.09**) were elevated, whereas albumin and cell counts were not significantly increased.”

3) In the section ‘CSF cell count, total protein, albumin, and albumin ratio’ under the heading ‘Affective disorders’:

“In the meta-analysis comparing to healthy controls, cell count was not significantly increased, whereas total protein levels (2 studies [53 patients]; SMD: **0.77**; 95% CI **0.36–1.18**, I^2^ = 0%), albumin (4 studies [**170** patients]; SMD = **0.26**; 95% CI **0.02–0.51**, I^2^ = 0) and albumin ratio were increased (4 studies [**302** patients]; SMD = **0.43**; 95% CI **0.25–0.61**, I^2^ = 0).”

was changed to:

“In the meta-analysis comparing to healthy controls, cell count was not significantly increased, whereas total protein levels (2 studies [53 patients]; SMD: **0.80**; 95% CI **0.39–1.21**, *I*^2^ = 0%), albumin (4 studies [**181** patients]; SMD = **0.28**; 95% CI **0.04–0.52**, *I*^2^ = 0) and albumin ratio were increased (4 studies [**298** patients]; SMD = **0.41**; 95% CI **0.23–0.60**, *I*^2^ = 0).”

4) In the section ‘CSF immunoglobulins’, under the heading ‘Schizophrenia spectrum disorders’:

“In the meta-analysis comparing to healthy controls, IgG/albumin ratio was decreased (1 study [32 patients]; SMD = −0.62; 95% CI −1.13 to −0.12), IgG ratio was increased (1 study [54 patients]; SMD = **0.60**; 95% CI **0.23–0.98**), whereas IgG levels and the IgG index were not significantly increased.”

was changed to:

“In the meta-analysis comparing to healthy controls, IgG/albumin ratio was decreased (1 study [32 patients]; SMD = −0.62; 95% CI −1.13 to −0.12), IgG ratio was increased (1 study [54 patients]; SMD = **0.68**; 95% CI **0.30–1.06**), whereas IgG levels and the IgG index were not significantly increased.”

5) In the section ‘CSF interleukins’, under the heading ‘Schizophrenia spectrum disorders’:

“In the meta-analysis comparing to healthy controls, IL-8 (3 studies [95 patients]; SMD = 0.46; 95% CI 0.17–0.75; I^2^ = 0%) and IL-6 (7 studies [230 patients]; SMD = **0.38**; 95% CI **0.02–0.74**; I^2^ = **64%**) were significantly increased. In a post-hoc analysis, we found that IL-6 was **only** significantly elevated in acute psychosis (SMD = 0.46; 95% CI **0.03–0.90**; I^2^ = **55%**) **but not in** chronic psychosis (SMD = **−0.08**; 95% CI **−0.77 to 0.60**; I^2^ = **64%**) with the between-group difference being not significant (p = **0.19**) (eFigure 3). The levels of IL-1alpha, IL-1beta and IL-2 were not statistically different from healthy controls.”

was changed to:

“In the meta-analysis comparing to healthy controls, IL-8 (3 studies [95 patients]; SMD = 0.46; 95% CI 0.17–0.75; *I*^2^ = 0%) and IL-6 (7 studies [230 patients]; SMD = **0.55**; 95% CI **0.35–0.76**; *I*^2^ = **1%**) were significantly increased. In a post-hoc analysis, we found that IL-6 was significantly elevated in acute psychosis (SMD = 0.46; 95% CI **0.22–0.71**; *I*^2^ = **1%**) **and** chronic psychosis (SMD = **−0.75**; 95% CI **0.39 to 1.12**; *I*^2^ = **0%**) with the between-group difference being not significant (*p* = **0.20**) (eFigure 3). The levels of IL-1alpha, IL-1beta and IL-2 were not statistically different from healthy controls.”

6) A number of changes were also made to Table 2 in the original article. The original, incorrect version of Table 2 is displayed below. Values which have now been corrected in the updated, original article are underlined. Additional rows have also been added to Table 2 in the updated, original article. These rows are ‘IL-1 alpha’ and ‘IL-2’.

**Table 2:** CSF immune related markers in patients with schizophrenia spectrum or affective disorders compared to healthy controls

Schizophrenia vs healthy controls							
CSF Marker	Studies	Cases	Control	SMD	95% CI	*p*-value	*I* ^2^
Cell count	1	32	31	0.19	−0.31 to 0.68	0.46	NA
Total protein	3	97	142	**0.38**	0.12 to 0.65	**0.004**	0%
Albumin	2	86	91	0.21	−0.29 to 0.70	0.41	62%
Albumin ratio	1	54	60	**0.62**	0.24 to 1.00	**0.001**	NA
IgG	2	86	91	−0.12	−0.93 to 0.69	0.77	85%
IgG Ratio	1	54	60	**0.60**	0.23 to 0.98	**0.002**	NA
IgG/Albumin ratio	1	32	31	**−0.62**	−1.13 to −0.12	**0.02**	NA
IgG Index	2	100	80	0.25	−0.07 to 0.56	0.13	7%
IL-1 Beta	2	40	39	0.67	−2.58 to 3.92	0.69	97%
IL-6	7	230	167	**0.38**	0.02 to 0.74	**0.04**	64%
IL-6R	1	46	35	−0.24	−0.68 to 0.20	0.28	NA
IL-8	3	95	102	**0.46**	0.17 to 0.75	**0.002**	0%
Neopterin	1	11	10	−0.05	−0.91 to 0.81	0.91	NA
MIP-1 alfa	1	8	8	−0.70	−1.72 to 0.32	0.18	NA
C3	1	46	35	0.00	−0.44 to 0.44	1.00	NA
MCP-2	1	46	35	0.21	−0.23 to 0.65	0.36	NA
TNFR2	1	46	35	0.06	−0.38 to 0.50	0.78	NA
TGFB1	1	44	19	0.29	−0.25 to 0.83	0.29	NA
TGFB2	1	44	19	−0.14	−0.68 to 0.40	0.61	NA

Affective Disorders vs healthy controls							
CSF Marker	Studies	Cases	Control	Mean ES	95% CI	*p*−value	*I* ^2^
Cell count	1	29	31	0.40	−0.11 to 0.91	0.13	NA
Total protein	2	53	48	**0.77**	0.36 to 1.18	**0.0002**	0%
Albumin	4	170	121	**0.26**	0.02 to 0.51	**0.04**	0%
Albumin ratio	4	302	238	**0.43**	0.25 to 0.61	**<0.00001**	0%
IgG	2	36	42	−0.22	−0.75 to 0.32	0.43	0%
IgG Ratio	1	29	11	0.33	−0.37 to 1.02	0.36	NA
IgG/Albumin ratio	1	7	31	−0.56	−1.39 to 0.28	0.19	NA
IgG Index	1	29	11	0.22	−0.48 to 0.91	0.54	NA
IL-1	1	18	25	0.61	−0.01 to 1.23	0.05	NA
IL-1 Beta	2	62	77	6.46	−7.48 to 20.39	0.36	99%
IL-6	7	159	242	0.40	−0.23 to 1.03	0.22	88%
IL-8	5	273	263	0.29	−0.15 to 0.73	0.19	82%
TNF-alpha	2	50	72	0.74	−0.46 to 1.93	0.23	89%
Eotaxin-1	1	75	43	−0.33	−0.77 to 0.04	0.08	NA
IP-10	2	75	43	−0.17	−0.55 to 0.20	0.37	NA
MIP-1B	1	75	43	−0.26	−0.64 to 0.12	0.17	NA
MCP-1	1	75	43	−0.28	−0.65 to 0.10	0.15	NA
MCP-4	1	75	43	**−0.76**	−1.15 to −0.37	**0.0001**	NA
TARC	1	75	43	**−0.57**	−0.95 to −0.19	**0.004**	NA

7) Fig. 2 has also been updated in the original article. The original, incorrect version of Fig. 2 is displayed below:

**Figure 1:**
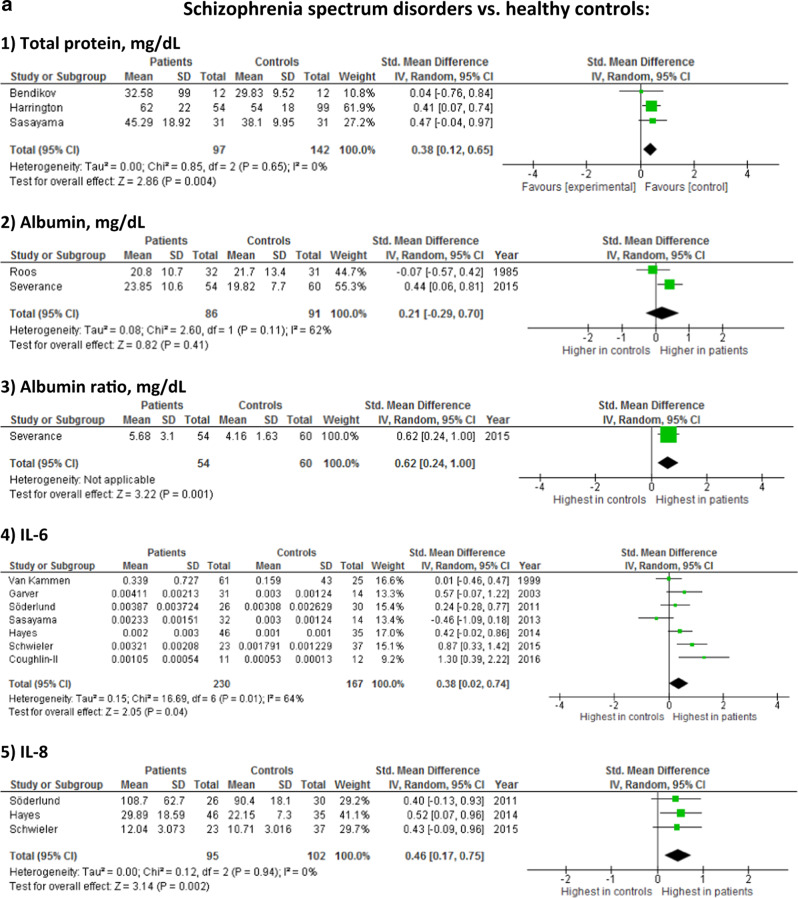

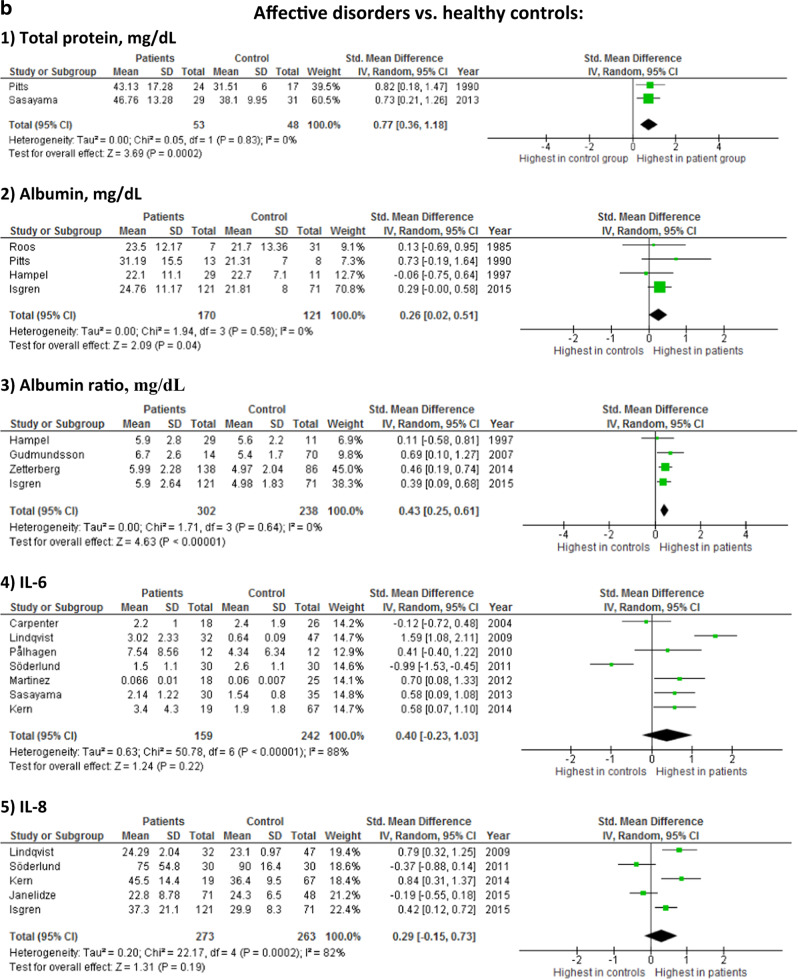
Fig. 2

8) The supplementary material has also now been replaced in the original article. All supplementary figures were updated for minor inconsistencies. The main change based on this was the post-hoc analysis of IL-6 levels among acute versus chronic schizophrenia patients. The results now show significantly increased IL-6 levels among both groups compared to healthy controls. The previous analyses only showed increased levels among patients with acute schizophrenia.

9) This article was also originally published under standard licence, but has now been made available under a [CC BY 4.0] licence.

The authors would like to apologise for these errors. This has been corrected in both the PDF and HTML versions of the Article.

