# Supplementary material for: Cerebrospinal fluid markers of inflammation and infections in schizophrenia and affective disorders: a systematic review and meta-analysis
Source: Mol Psychiatry. 2018 Aug 16;24(6):869–87. doi: 10.1038/s41380-018-0220-4 (PMC6756288; doi:10.1038/s41380-018-0220-4)
Supplement: Supplementary file 1 — Supplemental material [file 41380_2018_220_MOESM1_ESM.docx]

**Supplementary online content**

**eMethods.**

**eResults.**

**eTable 1. Results on investigated markers of CSF inflammation and infection.**

**eTable 2. Baseline characteristics and results from studies with somatic control subjects.**

**eTable 3. Baseline characteristics and results from studies without control subjects.**

**eTable 4. Bias assessment of studies included in the meta-analysis according to the Newcastle-Ottawa quality assessment scale for**

**case control studies.**

**eFigure 1. Forest plots on the results from studies investigating CSF immune-related markers in patients with schizophrenia spectrum or affective disorders (i.e. all the remaining forest plots that were not shown in Figure 2 in the main material).**

**eFigure 2. Forest plots on the results from studies published after the year 2000 investigating CSF immune-related markers in patients with schizophrenia spectrum or affective disorders.**

**eFigure 3. Post-hoc analyses comparing patients with acute psychosis or chronic psychosis, which was only possible for IL-6.**

**eReferences.**

**Prospero-protocol**

**eMethods**

**Assessment of study quality**

We assessed the quality of the included studies by using the Newcastle-Ottawa Scale (NOS) for case-control studies. This method assesses selection of cases and controls, comparability of cases and controls and ascertainment of exposure. For clarity we will specify adequacy for non-self explanatory items in the current study. Selection of cases was considered adequate if they were diagnosed by a valid diagnostic system (e.g. ICD or DSM), cases were considered adequate if they were recruited consecutively and less than 10% of cases refused to participate, adequate definition of controls also required lack of fulfillment of diagnostic criteria using the same diagnostic system used for cases. For adequacy of comparability, cases and controls should match on age and/or body mass index. In the current study CSF biomarker assessment was considered as the exposure. Studies were considered adequate in the Exposure item if the biomarker CSF levels were assessed blinded to case/control status. The third item in ‘Exposure’ was not relevant in the current study and therefore not assessed.

**eResults**

**Findings from studies combining cases with psychosis, schizophrenia and affective disorders**

A number of studies had combined groups of cases with psychosis, schizophrenia and affective disorders. Cell count was reported to be increased in 9.5% of the cases (1). The albumin ratio was increased in 29% of the cases (1) while another study did not find a difference between cases and neurological controls (2). One study found normal levels of IgG, IgM and IgA in cases (1) and another did not find a difference in levels of CSF/Serum ratios of IgG, IgM or IgA between cases and neurological controls (2). Oligoclonal bands in CSF were detected in up to 5.9% of cases (1,2) and an intrathecal immune response in 14% of cases (1). One study found a marginal elevation of IFN-gamma, TNF-alpha, IL-1beta, IL-2, IL-4, IL-5, IL-8, IL-10, IL12p70, IL-13 and IL-17 (3). Interferon was not detected in CSF of either cases or healthy controls (4). One study found antibodies against CMV in 13.8% of cases (4).

**Correlation between serum and CSF findings**

Studies found a significant correlation between serum/plasma and CSF levels of IgA (5), IgM (5), sIL-6r (6), IL-6 (7), eotaxin (8), IP-10 (8), and MIP-1 beta (8) while others did not find a correlation between serum and CSF levels of albumin (6,9), IgG (5,10), lymphocyte activational stage (11), the proportion of CD4+ or CD8+ T lymphocytes, CD4+/CD8+ ratio (12), IL-1beta (13), sIL-2r (14), IL-6 (15) or interferon (4).

**eTable 1. Results on investigated markers of CSF inflammation and infection.**

|  |  | **Patient** |  |  | **Control** |  |  | **95% CI** |  | **Marker** | **Disorder** |
| --- | --- | --- | --- | --- | --- | --- | --- | --- | --- | --- | --- |
| **Study** | **N** | **Mean** | **SD** | **N** | **Mean** | **SD** | **Effect size SMD** | **Lower** | **Upper** |  |  |
| **SCHIZOPHRENIA**  **SPECTRUM** |  |  |  |  |  |  |  |  |  |  |  |
| Harrington, 1985^1^ | 54 | 62 | 22 | 99 | 54 | 18 | 0.41 | 0.07 | 0.74 | Total protein, mg/dL | Schizophrenia |
| Bendikov, 2007^2^ | 12 | 32.58 | 9.90 | 12 | 29.83 | 9.52 | 0.27 | -0.53 | 1.08 | Total protein, mg/dL | Schizophrenia |
| Sasayama, 2013^3^ | 31 | 45.29 | 18.92 | 31 | 38.1 | 9.95 | 0.47 | -0.04 | 0.97 | Total protein, mg/dL | Schizophrenia |
| Sasayama, 2013^3^ | 32 | 3.41 | 2.33 | 31 | 2.97 | 2.32 | 0.19 | -0.31 | 0.68 | Cell count | Schizophrenia |
| Roos, 1985^4^ | 32 | 20.8 | 10.7 | 31 | 21.7 | 13.4 | -0.07 | -0.57 | 0.42 | Albumin, mg/dL | Schizophrenia |
| Severance, 2015^5^ | 54 | 23.85 | 10.6 | 60 | 19.82 | 7.7 | 0.44 | 0.06 | 0.81 | Albumin, mg/dL | Schizophrenia |
| Severance, 2015^5^ | 54 | 5.48 | 2.05 | 60 | 4.16 | 1.63 | 0.71 | 0.33 | 1.09 | Qalb | Schizophrenia |
| Roos, 1985^4^ | 32 | 3.5 | 1.58 | 31 | 5.3 | 4.29 | -0.55 | -1.06 | -0.05 | IgG, mg/dL | Schizophrenia |
| Severance, 2015^5^ | 54 | 2.65 | 1.32 | 60 | 2.35 | 0.85 | 0.27 | -0.10 | 0.64 | IgG, mg/dL | Schizophrenia |
| Severance, 2015^5^ | 54 | 2.63 | 1.2 | 60 | 1.95 | 0.77 | 0.68 | 0.30 | 1.06 | IgG ratio | Schizophrenia |
| Roos, 1985^4^ | 32 | 0.18 | 0.06 | 31 | 0.26 | 0.17 | -0.62 | -1.13 | -0.12 | IgG-Albumin ratio | Schizophrenia |
| Kirch, 1992^6^ | 46 | 0.54 | 0.24 | 20 | 0.44 | 0.1 | 0.47 | -0.06 | 1.01 | IgG index | Schizophrenia |
| Severance, 2015^5^ | 54 | 0.48 | 0.07 | 60 | 0.47 | 0.08 | 0.13 | -0.24 | 0.50 | IgG index | Schizophrenia |
| Rapaport, 1997^7^ | 60 | 0.96 | 0.64 | 21 | 1.31 | 0.56 | -0.56 | -1.06 | -0.05 | IL-1 alpha ng/mL | Schizophrenia |
| El-Mallakh, 1993^8^ | 2 | 0.19 | 0.135 | 2 | 0.105 | 0.002 | 0.51 | -2.97 | 3.99 | IL-1 alpha ng/mL | Schizophrenia/  schizoaffective |
| Licino, 1993^9^ | 10 | 0.445 | 0.308 | 10 | 0.342 | 0.193 | 0.38 | -0.50 | 1.27 | IL-1 alpha ng/mL | Schizophrenia |
| Katila, 1994^10^ | 14 | 17.9 | 1 | 9 | 25.9 | 4.6 | -2.62 | -3.80 | -1.44 | IL-1 beta pg/mL | Schizophrenia |
| Soderlund, 2009^11^ | 26 | 6.51 | 3.57 | 30 | 0.79 | 0.22 | 2.32 | 1.63 | 3.01 | IL-1 beta pg/mL | Schizophrenia |
| El-Mallakh, 1993^8^ | 27 | 0.459 | 0.19 | 11 | 0.416 | 0.2 | 0.22 | -0.48 | 0.92 | IL-2 ng/mL | Schizophrenia/  schizoaffective |
| Licino, 1993^9^ | 10 | 0.987 | 0.77 | 10 | 0.447 | 0.244 | 0.91 | -0.03 | 1.84 | IL-2 ng/mL | Schizophrenia |
| Rapaport, 1997^7^ | 60 | 1.03 | 0.64 | 21 | 1.25 | 0.76 | -0.32 | -0.82 | 0.18 | IL-2 ng/mL | Schizophrenia |
| Van Kammen, 1999^12^ | 61 | 0.53 | 0.7 | 25 | 0.25 | 0.43 | 0.44 | -0.03 | 0.91 | IL-6 ng/mL | Schizophrenia/  schizoaffective |
| Garver, 2003^13^ | 31 | 0.00411 | 0.00213 | 14 | 0.003 | 0.00124 | 0.57 | -0.07 | 1.22 | IL-6 ng/mL | Schizophrenia |
| Soderlund, 2009^11^ | 26 | 0.00387 | 0.00346 | 30 | 0.00308 | 0.002629 | 0.26 | -0.27 | 0.78 | IL-6 ng/mL | Schizophrenia |
| Sasayama, 2013^3^ | 32 | 0.00233 | 0.00151 | 35 | 0.00154 | 0.0008 | 0.65 | 0.16 | 1.15 | IL-6 ng/mL | Schizophrenia |
| Hayes, 2014^14^ | 46 | 0.002165 | 0.00352 | 35 | 0.001120 | 0.000644 | 0.38 | -0.06 | 0.83 | IL-6 ng/mL | Schizophrenia |
| Schwieler, 2015^15^ | 23 | 0.00321 | 0.00208 | 37 | 0.001791 | 0.001229 | 0.87 | 0.33 | 1.42 | IL-6 ng/mL | Schizophrenia/  schizoaffective |
| Coughlin, 2016^16^ | 11 | 0.00105 | 0.00054 | 12 | 0.00053 | 0.00013 | 1.30 | 0.39 | 2.22 | IL-6 ng/mL | Schizophrenia |
| Hayes, 2014^14^ | 46 | 0.879 | 0.271 | 35 | 0.946 | 0.278 | -0.24 | -0.68 | 0.20 | IL-6R ng/mL | Schizophrenia |
| Soderlund, 2009^11^ | 26 | 108.7 | 62.7 | 30 | 90.4 | 18.1 | 0.40 | -0.13 | 0.93 | IL-8 pg/mL | Schizophrenia |
| Hayes, 2014^14^ | 46 | 29.89 | 18.59 | 35 | 22.15 | 7.3 | 0.52 | 0.07 | 0.96 | IL-8 pg/mL | Schizophrenia |
| Schwieler, 2015^15^ | 23 | 12.04 | 3.073 | 37 | 10.71 | 3.016 | 0.43 | -0.09 | 0.96 | IL-8 pg/mL | Schizophrenia/  schizoaffective |
| Nikkilä, 2002^17^ | 8 | 4.7 | 0.3 | 8 | 5.1 | 0.7 | -0.70 | -1.72 | 0.32 | MIP-1 alpha pg/mL | Schizophrenia |
| Hayes, 2014^14^ | 46 | 0.0021 | 0.0009 | 35 | 0.0021 | 0.0007 | 0.00 | -0.44 | 0.44 | C3 mg/mL | Schizophrenia |
| Hayes, 2014^14^ | 46 | 4.33 | 10.23 | 35 | 2.27 | 0.93 | 0.26 | -0.18 | 0.71 | MCP-2 pg/mL | Schizophrenia |
| Hayes, 2014^14^ | 46 | 0.48 | 0.17 | 35 | 0.47 | 0.14 | 0.06 | -0.38 | 0.50 | TNFR2 ng/mL | Schizophrenia |
| Vawter, 1997^18^ | 44 | 29.9 | 13.93 | 19 | 26.0 | 11.77 | 0.29 | -0.25 | 0.83 | TGF-beta1 pg/mL | Schizophrenia |
| Vawter, 1997^18^ | 44 | 219.9 | 70.3 | 19 | 230.1 | 73.23 | -0.14 | -0.68 | 0.40 | TGF-beta2 pg/mL | Schizophrenia |
| Nikkilä, 2002^17^ | 11 | 2.8 | 2.2 | 10 | 2.9 | 1.5 | -0.05 | -0.91 | 0.81 | Neopterin nmol/L | Schizophrenia |
|  |  |  |  |  |  |  |  |  |  |  |  |
|  |  | **Patient** |  |  | **Control** |  |  | **95% CI** |  |  |  |
| **AFFECTIVE**  **DISORDERS** | **N** | **Mean** | **SD** | **N** | **Mean** | **SD** | **Effect size SMD** | **Lower** | **Upper** | **Marker** | **Disorder** |
| Sasayama, 2013^3^ | 29 | 4.03 | 2.91 | 31 | 2.97 | 2.32 | 0.40 | -0.11 | 0.91 | Cell count | MDD |
| Pitts, 1990^19^ | 24 | 44.67 | 18.04 | 17 | 31.51 | 6 | 0.90 | 0.24 | 1.55 | Total protein mg/dL | MDD, depression NOS, bipolar disorder, schizoaffective disorder and drug dep D/O |
| Sasayama, 2013^3^ | 29 | 46.76 | 13.28 | 31 | 38.10 | 9.95 | 0.73 | 0.21 | 1.26 | Total protein mg/dL | MDD |
| Roos, 1985^4^ | 7 | 23.5 | 12.17 | 31 | 21.7 | 13.36 | 0.13 | -0.69 | 0.95 | Albumin mg/dL | Depression |
| Pitts, 1990^19^ | 24 | 28.44 | 14.23 | 17 | 20.96 | 6.85 | 0.62 | -0.01 | 1.26 | Albumin mg/dL | MDD, depression NOS, bipolar disorder, schizoaffective disorder and drug dep D/O |
| Hampel, 1997^20^ | 29 | 22.1 | 11.1 | 11 | 22.7 | 7.1 | -0.06 | -0.75 | 0.64 | Albumin mg/dL | MDD |
| Isgren, 2015^21^ | 121 | 24.760 | 11.17 | 71 | 21.81 | 8 | 0.29 | -0.00 | 0.58 | Albumin mg/dL | bipolar spectrum disorder (type 1+2+NOS) |
| Gudmundsson, 2007^22^ | 14 | 6.7 | 2.6 | 70 | 5.4 | 1.7 | 0.69 | 0.10 | 1.27 | Qalb | MDD and dysthymia |
| Hampel, 1997^20^ | 29 | 5.9 | 2.8 | 11 | 5.6 | 2.2 | 0.11 | -0.58 | 0.81 | Qalb | MDD |
| Zetterberg, 2014^23^ | 134 | 5.89 | 2.22 | 86 | 4.97 | 2.04 | 0.43 | 0.15 | 0.70 | Qalb | Bipolar disorder type I, II, NOS, cyclothymia, or schizo-affective syndrome manic type |
| Isgren, 2015^21^ | 121 | 5.9 | 2.64 | 71 | 4.98 | 1.83 | 0.39 | 0.09 | 0.68 | Qalb | Bipolar spectrum disorder (type 1+2+NOS) |
| Roos, 1985^4^ | 7 | 3.92 | 2.22 | 31 | 5.3 | 4.28 | -0.34 | -1.16 | 0.49 | IgG mg/dL | Depression |
| Hampel, 1997^20^ | 29 | 2.92 | 1.54 | 11 | 3.11 | 1.1 | -0.13 | -0.82 | 0.57 | IgG mg/dL | MDD |
| Hampel, 1997^20^ | 29 | 2.8 | 1.7 | 11 | 2.3 | 0.7 | 0.33 | -0.37 | 1.02 | IgG ratio | MDD |
| Roos, 1985^4^ | 7 | 0.17 | 0.08 | 31 | 0.26 | 0.17 | -0.56 | -1.39 | 0.28 | IgG-Albumin ratio | Depression |
| Hampel, 1999^24^ | 29 | 0.46 | 0.07 | 11 | 0.44 | 0.13 | 0.22 | -0.48 | 0.91 | IgG index | MDD |
| Hampel, 1999^24^ | 29 | 1 |  | 11 | 0 |  |  |  |  | OCB | MDD |
| Martinez, 2012^25^ | 18 | 0.073 | 0.022 | 25 | 0.064 | 0.004 | 0.61 | -0.01 | 1.23 | IL-1 pg/mL | MDD |
| Lindqvist, 2009^26^ | 32 | 0.064 | 0.041 | 47 | 0.07 | 0.07 | -0.10 | -0.55 | 0.35 | IL-1 beta pg/mL | MDD and depression NOS |
| Söderlund, 2011^27^ | 30 | 4.2 | 2.74 | 30 | 0.8 | 0.22 | 1.73 | 1.13 | 2.33 | IL-1 beta pg/mL | Bipolar |
| Carpenter, 2004^28^ | 18 | 2.2 | 1.0 | 26 | 2.4 | 1.9 | -0.12 | -0.72 | 0.48 | IL-6 pg/mL | Unipolar depression |
| Lindqvist, 2009^26^ | 32 | 3.02 | 9.21 | 47 | 0.64 | 0.62 | 0.40 | -0.05 | 0.86 | IL-6 pg/mL | MDD and depression NOS |
| Pålhagen, 2010^29^ | 12 | 7.54 | 8.56 | 12 | 4.34 | 6.34 | 0.41 | -0.40 | 1.22 | IL-6 pg/mL | Depression |
| Söderlund, 2011^27^ | 30 | 1.5 | 1.1 | 30 | 2.6 | 1.1 | -0.99 | -1.53 | -0.45 | IL-6 pg/mL | Bipolar |
| Martinez, 2012^25^ | 18 | 0.066 | 0.01 | 25 | 0.06 | 0.007 | 0.70 | 0.08 | 1.33 | IL-6 pg/mL | MDD |
| Sasayama, 2013^3^ | 30 | 2.14 | 1.22 | 35 | 1.54 | 0.80 | 0.58 | 0.09 | 1.08 | IL-6 pg/mL | MDD |
| Kern, 2014^30^ | 19 | 3.4 | 4.3 | 67 | 1.9 | 1.8 | 0.58 | 0.07 | 1.10 | IL-6 pg/mL | Major and minor depression (DSM-4) |
| Lindqvist, 2009^26^ | 32 | 24.29 | 7.24 | 47 | 23.1 | 6.65 | 0.17 | -0.28 | 0.62 | IL-8 pg/mL | MDD and depression NOS |
| Söderlund, 2011^27^ | 30 | 75 | 54.8 | 30 | 90 | 16.4 | -0.37 | -0.88 | 0.14 | IL-8 pg/mL | Bipolar |
| Kern, 2014^30^ | 19 | 45.5 | 14.4 | 67 | 36.4 | 9.5 | 0.84 | 0.31 | 1.37 | IL-8 pg/mL | Major and minor depression (DSM-4) |
| Isgren, 2015^21^ | 121 | 37.3 | 21.1 | 71 | 29.9 | 8.3 | 0.42 | 0.12 | 0.72 | IL-8 pg/mL | bipolar spectrum disorder (type 1+2+NOS) |
| Janelidze, 2015^3132^ | 71 | 22.79 | 8.78 | 48 | 24.3 | 6.5 | -0.19 | -0.56 | 0.18 | IL-8 pg/mL | Depression and dysthymia |
| Lindqvist, 2009^26^ | 32 | 0.15 | 0.051 | 47 | 0.13 | 0.07 | 0.31 | -0.14 | 0.77 | TNF-alpha pg/mL | MDD and depression NOS |
| Martinez, 2012^25^ | 18 | 0.105 | 0.113 | 25 | 0.095 | 0.057 | 0.12 | -0.49 | 0.72 | TNF-alpha pg/mL | MDD |
| Janelidze, 2013^31^ | 75 | 14.55 | 9 | 43 | 17.2 | 5.3 | -0.33 | -0.71 | 0.04 | Eotaxin-1 pg/mL | MDD, dysthymia, depression NOS and bipolar type I (DSM-3-R) |
| Janelidze, 2013^31^ | 75 | 176.04 | 142.3 | 43 | 200.9 | 147.3 | -0.17 | -0.55 | 0.20 | IP-10 (interferon gamma-induced protein-10) pg/mL | MDD, dysthymia, depression NOS and bipolar type I (DSM-3-R) |
| Janelidze, 2013^31^ | 75 | 33.49 | 24.11 | 43 | 39.7 | 22.8 | -0.26 | -0.64 | 0.12 | MIP-1 beta (macrophage inflammatory protein-1 beta) pg/mL | MDD, dysthymia, depression NOS and bipolar type I (DSM-3-R) |
| Janelidze, 2013^31^ | 75 | 801.82 | 404.18 | 43 | 903.6 | 279 | -0.28 | -0.65 | 0.10 | MCP-1 (monocyte chemotactic protein-1) pg/mL | MDD, dysthymia, depression NOS and bipolar type I (DSM-3-R) |
| Janelidze, 2013^31^ | 75 | 4.32 | 2.29 | 43 | 6 | 2 | -0.76 | -1.15 | -0.37 | MCP-4 (monocyte chemotactic protein-4) pg/mL | MDD, dysthymia, depression NOS and bipolar type I (DSM-3-R) |
| Janelidze, 2013^31^ | 75 | 4.212 | 2.33 | 43 | 5.5 | 2.1 | -0.57 | -0.95 | -0.19 | TARC (thymus and activation regulated chemokine) pg/mL | MDD, dysthymia, depression NOS and bipolar type I (DSM-3-R) |

| **eTable 2. Baseline characteristics and results from studies with somatic control subjects.** | | | | | | |
| --- | --- | --- | --- | --- | --- | --- |
| **Schizophrenia spectrum disorders** | | | | | | |
| **Study** | **Case subjects** | | | **Control subjects** | | **Results** |
|  | **N, diagnosis (diagnostic tool)** | **No. (%) of Males/Mean Age, y** | **Medication status** | **N, type** | **No. (%) of Males/Mean Age, y** |  |
| ^i^Hoerster, 1963 (16) | 306, schizophrenia (NA) | NA/NA | NA | 30, non-psychotic patients with neurotic or personality disorders | NA/NA | ↔Total protein, albumin, alpha1-, alpha2-, beta-1 and beta2-globulin: no difference between cases and controls  ↑Gamma-globulin in cases compared with controls |
| Selecki, 1964 (17) | 31, schizophrenia (NA) | 10 (32)/41.7 | NA | 47, epilepsy  25, epilepsy with congenital mental defects | 27 (57) and  14 (56)  /43.1 and 33.7 | ↔Total protein: no difference between cases and controls  ↓Albumin-fraction out of total protein in cases compared with controls (p<0.01)  ↑Beta-globulin fraction out of total protein in cases compared with controls (p<0.05) |
| Shanmugam, 1971 (18) | 30, schizophrenia (NA) | NA/ Range 28-50 | NA | 24, surgical | NA/ Range 19-42 | ↔Total protein, gamma-, alpha1- and alpha2-globulin fractions out of total protein: no difference between cases and controls  ↓Albumin-fraction out of total protein compared with controls  ↑Beta1- and Beta2-globulin fractions out of total protein in cases compared with controls |
| Bock, 1978 (19) | 17, schizophrenia | 12 (71)/27 | No medication for >1 month | 22, psychiatric and neurological | NA/36 | Neg. test for syphilis in cases.  CSF/serum IgG ratio (mean (SEM)): 2.6 (0.37) in schizophrenia, 2.6 (0.20) in controls.  CSF/serum IgA ratio (mean (SEM)): 0.7 (0.23) in schizophrenia, 0.9 (0.12) in controls.  CSF/serum pre-albumin ratio (mean (SEM)): 65 (4.9) in schizophrenia, 72 (4.6) in controls.  CSF/serum transferrin ratio (mean (SEM)): 6.7 (0.60) in schizophrenia, 6.6 (0.52) in controls.  CSF/serum albumin ratio (mean (SEM)): 4.9 (0.63) in schizophrenia, 4.8 (0.42) in controls.  CSF/serum alpha2-macroglobulin ratio (mean (SEM)): 0.3 (0.13) in schizophrenia, 0.2 (0.09) in controls. |
| Rimon, 1978 (20) | 12, schizophrenia (Diagnostic criteria of Carpenter et al.) | NA/NA | No antipsychotic medication ≥ 2 weeks | 10, surgical | NA/NA | Cell count, total protein: normal in cases  Measles antibodies not present in cases or controls  HSV-1 and rubella antibodies: no difference between cases and controls |
| ^ii^Torrey, 1978 (10) | 66, schizophrenia, schizoaffective psychosis and manic-depressive psychosis (diagnostic criteria of Carpenter et al. and Spitzer et al.) | 28 (42)/26.8 | 48 cases did not receive psychotropic medication ≥ 2 weeks | 10, surgical  80, neurological | 8 (80)/47 | IgG/total protein%: no difference between cases and controls  ↑IgA/total protein% in cases with schizophrenia with multiple admissions compared with controls  Measles antibodies detected in two-thirds and HSV-1 antibodies in over one-third of the cases  CMV antibodies in 0 cases and 1 control  CSF/serum measles antibodie <160 in 4 cases (6.1%) and in none of the controls indicative of BBB disturbance or intrathecal antibody synthesis  ↔No correlation between serum and CSF IgG or between IgG/total protein% or IgA/total protein% and psychosis rating (Bunney and Hamburg 1963) |
| Crow, 1979 (21) | 47, schizophrenia (diagnostic criteria of Feighner et al.) | 26 (55)/NA | NA | 9, neurological, surgical and persons being subjected to spinal anesthesia | NA | Virus-like agent in 18 cases (38%) |
| Albrecht, 1980 (22) | 60, schizophrenia and schizoaffective psychosis (except 2 cases with no information on diagnosis) (NA) | 38 (63)/32.5 | 53 cases received antipsychotic medication | 26; 12 healthy and 14 non-schizophrenic in-patients in a drug rehabilitation programme | 18 (69)/30.8 | ↔Albumin, albumin ratio, IgG, IgG CSF/serum ratio, antibody titers for CMV, vaccinia, HSV-1 and influenza: no difference between cases and controls  ↑CSF/serum ratio for CMV, vaccinia and influenza-antibody titres in cases compared with controls |
| Delisi, 1981 (5) | 35, schizophrenia (diagnostic criteria of Spitzer a al., 1977) | 27 (77)/28.7 | No psychotropic medication ≥ 3 weeks | 16, neurological | 13 (81)/38.3 | ↓IgG, IgA, IgM, IgA/total protein% (p>0.001), and IgM/total protein% (p>0.005) in cases compared with controls  ↔IgG/TP%: no difference between cases and controls  Correlation between plasma and CSF IgA and IgM  ↔No correlation between plasma and CSF IgG or between antipsychotic medication and immunoglobulins |
| ^i,iii^Gotlieb-Stematsky, 1981 (23) | 41, schizophrenia (diagnostic criteria of Feighner et al.) | 16 (39)/34 | NA | 25, neurological | 10 (40)/43.7 | ↑Total protein in 3/19 cases (15.8%) and 11/20 controls (55%)  HSV-1 antibodies present in 11/18 cases (61.1%) and 8/20 controls (40%) ↓CSF/serum HSV-1 antibody ratio in 3 cases and 0 controls  EBV-viral capsid antibodies present in 1/19 case (5.3%) and 1/20 control (5%)  CMV antibodies not present in cases or controls  Measles antibodies present in 1/19 case (5.3%) and 2/20 controls (10%) |
| Pandey, 1981 (24) | 54, schizophrenia (DSM-3) | 33 (61)/ Range 17-50 | No psychotropic medication for 3 months | NA, medical | Range 17-50 | Anti-brain antibodies present in 26 cases (48.1%) and 0 controls |
| Leonardi, 1982 (25) | 25, schizophrenia | 17 (68)/26 | All under neuroleptic treatment | 37 somatically ill drug-free controls | 15 (41)/38 | No difference in CSF IgG and albumin between schizophrenia and controls |
| ^i^Torrey, 1982 (26) | 178, schizophrenia (research diagnostic criteria) | 109 (61)/32.6 | 38 cases did not receive psychotropic medication | 10, surgical  16, healthy  14, patients in an in-patients drug-addiction program  1, volunteer from the National Institutes of Health | 30 (73)/34.0 | ↔CMV IgG, IgA and IgM investigated in 109 cases and 24 controls: no difference between cases and controls in percentage of pos. samples or mean antibody titer; no detection of CMV antigen  ↑CMV IgM (investigated in all cases and controls) in cases (detected in 20 cases (11%)) compared with controls (detected in 0 controls) (p=0.025)  Investigation of albumin ratio in one-third of cases: normal  ↔No correlation between CMV IgM and psychotropic medication |
| Rimon, 1983 (27) | 15, schizophrenia (diagnostic criteria of Carpenter et al. and Feighner et al.) | 9 (60)/35.3 | No antipsychotic treatment ≥ 2 weeks | 15, spinal anesthesia in connection with diagnostic X-ray or urological examination | 10 (67)/39.8 | Cell count, total protein: normal in cases  Interferon not present in cases or controls  ↔Myelin basic protein- and glial fibrillary acidic protein-antibodies: no difference between cases and controls |
| Torrey, 1983 (28) | 178, schizophrenia (research diagnostic criteria) | 109 (61)/32.6 | 38 cases did not receive psychotropic medication | 10, surgical  16, healthy  14, patients in an in-patients drug-addiction program  1, volunteer from the National Institutes of Health | 30 (73)/34.0 | Albumin, IgG, BBB permeability: normal in cases (N=60) and (N=26) controls  CSF/serum measles antibody titer: very high in 1/60 case  ↑Influenza A and vaccinia antibodies in cases (N=60) compared with controls (N=26)  Neg. antibodies against mumps (N=60 cases, 26 controls), rubella (N=60 cases, 26 controls), parvovirus (N=38 cases), adenovirus (N=109 cases, 26 controls), hepatitis B (N=30 cases), HSV-1 (N=178 cases, 41 controls), EBV (N=109 cases, 24 controls), mycoplasma (N=20 cases), toxoplasma gondii (N=38 cases) |
| Tiwari, 1984 (29) | 30, schizophrenia (diagnostic criteria of Feighner et al.) | NA/32 | All cases received barbiturates | 40, surgical and neurological | NA/NA | IgG present in cases and controls  IgA and IgM present in 0 cases and 5 (12.5%) controls  IgG/total protein%: increased in cases compared with surgical controls (p<0.01) but decreased compared with neurological controls (p<0.01). |
| King, 1985 (30) | 17, schizophrenia (DSM-3) | 11 (65)/39 | NA | 17, surgical | 11 (65)/38.5 | Cell count normal in cases (<5/mL)  Total protein, CMV-, HSV-, VZV-, adeno-, rubella, and measles-antibodies, and CSF/serum ratio for CMV, HSV, VZV and adeno: no difference between cases and controls  ↓IgG, IgG CSF/serum ratio, antibody titers for mumps, CSF/serum ratio for measles, rubella and mumps in cases compared with controls |
| Roy, 1985 (31) | 16, schizophrenia (DSM-3) | 9 (56)/26 | No psychotropic medication ≥ 2 weeks | 11, depression and bipolar disorder (DSM-3); drug-free for at least 2 weeks | 10 (91)/NA | Interferon present in 1 case (6.25%) (with a recurrent urinary tract infection at the time of LP) and 0 controls (non-significant difference) |
| Shrikhande, 1985 (32) | 31, schizophrenia spectrum disorder, mania and psychosis (ICD-9) | NA/NA | As least 10 cases did not receive psychotropic medication ≥ 3 weeks | 10, surgical | NA/NA | CMV IgM not present in cases or controls  ↑CMV IgG in 1 case (3.1%) and 4 controls (40%) |
| Rimon, 1986 (33) | 40, schizophrenia (diagnostic criteria of Feighner et al.) | 30 (75)/40.8 | No antipsychotic medication ≥ 3 weeks | 40, neurological | 21 (52.5)/ 44.5 | CMV antibodies not present in cases or controls  ↑CSF/serum CMV antibody-ratio in 5 cases (17%) and 1 control (4%) |
| Bergquist, 1993 (34) | 20, schizophrenia and BPD with psychosis (DSM-3-R) | 17 (85)/35 | All cases received psychotropic medication | 22, neurological |  | Total protein, albumin, albumin ratio, IgG index: normal in cases and controls  ↑Dopamine IgG in cases (present in 100%) compared with controls (present in 41%) (p<0.001) |
| Srikanth, 1994 (35) | 35, schizophrenia, mania with psychosis and psychosis NOS (ICD-9) | 26 (74)/25.8 | No psychotropic medication for 6 months | 35, surgical | 25 (71)/29.4 | Mycobacterium tuberculosis, cysticercus cellulosae, HIV-1, CMV, measles and JEV-2 antibodies: not present in cases or controls  HSV-1, mumps antibodies: present in 1 case (2.9%) and 0 controls (diagnostic)  VZV antibodies: present in 2 cases (5.7%) and 0 controls (not diagnostic) |
| ^iv^Barak, 1995 (36) | 16, schizophrenia (DSM-3-R) | 16 (100)/42.7 | All cases received psychotropic medication | 10, neurological | 10 (100)/ 44.8 | Cell count normal in cases and controls (<3lymphocytes/mm^3^)  ↔IL-2, IL-6 and TNF-alpha: no difference between cases and controls  ↓IL-1beta, sIL-2R in cases compared with controls  ↔No correlation between CSF findings and psychotropic medication |
| Nikkilä, 1995 (12) | 31, schizophrenia (DSM-3-R) | 16 (52)/31.2 | All cases received psychotropic medication | 21, neurological | 6 (29)/43 | CD4+/CD8+ ratio and proportion of CD4+ and CD8+ T lymphocytes: no difference between cases and controls  Proportion of CD4+ T lymphocytes pooled together: 23 cases (74%) had abnormal CD4+ and/or CD8+ levels, both abnormally high and low  ↔No correlation between CSF and peripheral blood subset findings |
| Sierra-Honigmann, 1995 (37) | 48, schizophrenia (DSM-3-R) | NA/NA | All cases received antipsychotic medication | 18, monozygotic twins discordant for schizophrenia (9 sets) | 14 (78)/NA | CMV, HIV, Influenza A, BDV and BVDV: viral nucleic acid sequences not present in cases or controls |
| ^i^Deuschle, 1998 (38) | 27, schizophrenia (DSM-3-R) | 12 (44)/39 | NA | 102, neurological | 50 (49)/47.7 | Borna disease virus antibodies and antigens: not present in cases, present in respectively 1 (1.0%) and 2 controls (2.0%) |
| Nikkilä, 1999 (39) | 35, schizophrenia (DSM-3-R) | 19 (54)/33 | No antipsychotic medication ≥ 4 months | 46, neurological | 21 (46)/32.5 | ↔Cell count: no difference between cases and controls  Albumin ratio normal in cases  ↑Proportion cells morphologically classified as mononuclear phagocytes/macrophages in cases compared with controls  13 cases underwent a second LP after a weeks of treatment with anti-psychotic medication with a tendency towards normalization of the cytological picture |
| Karlsson, 2001 (40) | 55, schizophrenia and schizoaffective disorder (DSM-4) | NA/Range 18-65 | >20 cases received psychotropic medication | 12, undergoing spinal anesthesia  18, healthy | NA and 9 (50)/NA and range 21-46 | Nucleotide sequences homologous to those of known retroviruses in 11 cases (20%) and 0 controls |
| Nikkilä, 2001 (11) | 30, schizophrenia (DSM-3-R) | 13 (43)/31.1 | No antipsychotic medication ≥ 4 months | 46, neurological | 21 (46)/32.7 | Cell count, albumin ratio: normal in cases and controls  ↓Frequency of lymphocytes out of the total number of mononuclear cells in cases compared with controls  ↓Number of morphologically normal lymphocytes out of the total number of lymphocytes in cases compared with controls  ↑Number of morphologically activated lymphocytes out of the total number of lymphocytes in cases compared with controls  ↑Frequency of macrophages out of the total number of mononuclear cells in cases compared with controls  ↔No correlation between lymphocyte activational stage in peripheral blood and CSF, between lymphocyte activational stage and psychiatric symptoms (BPRS), or between antipsychotic medication and lymphocytic profile |
| Zhu, 2016 (41) | 22, schizophrenia (ICD-10) | 14 (64)/30.48 | No cases received antipsychotic medication | 10, surgical | 5 (50)/29.96 | ↓TNF–alpha in cases compared with controls (p<0.05)  ↔No correlation between TNF-alpha and psychiatric symptoms (PANSS) (p<0.05) |
| **Affective disorders** | | | | | | |
| **Study** | **Case subjects** | | | **Controls** |  | |
|  | **N, diagnosis (diagnostic tool)** | **No. (%) of Males/Mean Age, y** | **Medication status** | **N, type** | **No. (%) of Males/Mean Age, y** | **Results** |
| ^i^Hoerster, 1963 (16) | 42, affective disorder (NA) | NA/NA | NA | 30, non-psychotic patients with neurotic or personality disorders | NA/NA | Total protein, albumin, alpha1-, alpha2-, beta1-, beta2- and gamma-globulin: no difference between cases and controls |
| Bock, 1978 (19) | 14, depression (3 had previous manic episodes) | 8 (57)/46 | No medication for >1 month | 22, psychiatric and neurological | NA/36 | Neg. test for syphilis in cases.  CSF/serum IgG ratio (mean (SEM)): 3.0 (0.22) in depression, 2.6 (0.20) in controls.  CSF/serum IgA ratio (mean (SEM)): 1.2 (0.13) in depression, 0.9 (0.12) in controls.  CSF/serum pre-albumin ratio (mean (SEM)): 63 (3.1) in depression, 72 (4.6) in controls.  CSF/serum transferrin ratio (mean (SEM)): 7.0 (0.53) in depression, 6.6 (0.52) in controls.  CSF/serum albumin ratio (mean (SEM)): 5.5 (0.37) in depression, 4.8 (0.42) in controls.  CSF/serum alpha2-macroglobulin ratio (mean (SEM)): 0.8 (0.22) in depression, 0.2 (0.09) in controls. |
| ^i,v^Gotlieb-Stematsky, 1981 (23) | 27, affective disorders (diagnostic criteria of Feighner et al.) | 8 (30)/44 | NA | 25, neurological | 10 (40)/43.7 | Total protein: normal in cases, increased in 11 controls (55.0%)  HSV-1 antibodies: present in 4/10 cases (40.0%) and 8/20 controls (40.0%)  ↓CSF/serum HSV-1 antibody-ratio in 1 case and 0 controls  EBV viral capsid antibody: present in 3/10 cases (30.0%) and 1/20 controls (5.0%)  CMV antibodies: not present in cases or controls  MV antibodies: not present in cases, present in 2/20 controls (10.0%) |
| Leonardi, 1982 (25) | 22, endogenous depression | 8 (36)/55 | All treated w tricyclic antidepressants | 37 somatically ill drug-free controls | 15 (41)/38 | No difference in CSF IgG and albumin between depression and controls |
| ^i^Torrey, 1982 (26) | 17, bipolar disorder (NA) | 5 (29)/40.2 | NA | 10, surgical  16, healthy  14, patients in an in-patients drug-addiction program  1, volunteer from the National Institutes of Health | 30 (73)/34.0 | CMV IgM detected in 3 cases (18%) and 0 controls |
| Kumar, 1986 (42) | 30, depression (diagnostic criteria of Feighner et al.) | NA/40.5 | No cases received psychotropic medication | 20, surgical | NA/33.7 | Total protein: Increased in 11 cases (36.6%), normal in controls  ↑IgG in cases compared with controls (p<0.05) and present in all cases and controls  IgA: present in 7 cases (23.3%) and 0 controls  IgG/total protein%, mean (SD): 8.0 (2.6) in cases, 6.6 (1.9) in controls IgA/total protein%, mean (SD): 6.7 (0.5) in cases.  IgM: not present in cases or controls |
| Tiwari, 1990 (43) | 30, depression (diagnostic criteria of Feighner et al.) | NA/ Range 17-50 | No psychotropic medication for 15 days apart from barbiturates when required | 20, neurological  20, surgical | NA/NA | Total protein: increased in cases compared with surgical controls but decreased compared with neurological controls  CMV and rubella antibodies: not detected in cases or controls  IgG: present in all cases and controls  IgA: present in 7 cases (23.3%) and 5 neurological controls (25.0%)  IgM: present in 0 cases and 5 neurological cases (25.0%)  ↑IgG/total protein% in cases compared with surgical controls (p=0.05)  ↓IgA/total protein% compared with neurological controls (p=0.05) |
| ^i^Deuschle, 1998 (38) | 73, MDD and BPD (DSM-3-R) | 31 (42)/49 | NA | 102, neurological | 50 (49)/47.7 | Borna disease virus antibodies: present in 2 cases (3%) and 2 controls (2%)  Borna disease virus antigen: present in 3 cases (4%) and 1 control (1%) |
| Levine, 1999 (14) | 13, MDD and BPD (DSM-3-R) | 2 (15)/56 | No psychotropic medication ≥ 1 week for cases with depression | 10, neurological | 5 (50)/43 | Cell count, total protein: normal in cases and controls  ↓sIL-2R in cases compared with controls  ↔No correlation between serum and CSF sIL-2R |
| ^vi^Levine, 1999 (44) | 13, MDD and BPD (DSM-3-R) | 2 (15)/56 | No psychotropic medication ≥ 1 week for cases with depression | 10, neurological | 5 (50)/43 | ↑IL-1beta in cases compared with controls  ↓IL-6 in cases compared with controls  ↔TNF-apha: no difference between cases and controls or IL-1beta and psychiatric symptoms (HDS) |
| ^vii^Schuld, 2004 (45) | 22, MDD (DSM-4) | 13 (59)/47.4 | NA | 22, neurological | 13 (59)/33.3 | ↑Cell count in 3 cases (13.1%) and 2 controls (9.1%) (non-significant difference)  ↑Albumin ratio in 4 cases (18%) and 4 controls (18%) (non-significant difference)  Oligoclonal bands present in 4 cases (4%), no difference cases and controls |
| Stich, 2015 (46) | 40, bipolar (DSM-4) | 15 (38)/48.3 | 33 cases received psychotropic medication | 26, neurological | 3 (12)/42.8 | ↑Cell count in 3 cases (7.5%) and 2 controls (7.7%) (p>0.467); samples with cell counts>20µL were excluded  ↑Total protein in 35.0% cases and 30.8% controls, (p>0.467)  ↑Albumin ratio in 15.0% cases and 11.5% controls (p>0.467)  ↑IgG index in 4 cases (10%) (2 had previously been diagnosed with MS) and 0 controls (p=0.074)  IgG CSF/serum ratio: normal in cases and controls  Oligoclonal bands present in 5 cases (12.5%) (2 had previously been diagnosed with MS) and 0 controls (p=0.074)  Intrathecal immune response in 12 cases (30.0%) (p<0.008 in comparison to controls)  T. gondii antibodies: present and at a normal level in 21 cases (52.5%) and 8 controls (31%)  ↑T. gondii antibody index* (AI) in 5 cases (12.5%) and 1 control (3.8%) (p>0.216)  HSV-1+2 antibodies: present and at a normal level in 34 cases (85%) and 22 controls (84.6%)  ↑HSV-1+2 AI in 3 cases (7.5%) and 0 controls (p>0.216)  CMV antibodies present and at a normal level in 20 cases (50%) and 16 controls (61.5%)  ↑CMV AI in 1 case (2.5%) and 0 controls (p>0.216)  EBV antibodies: present and at a normal level in 24 cases (60%) and 12 controls (46.1%)  ↑EBV AI in 1 case (2.5%) and 0 controls (p>0.216)  *An increased antibody index indicates intrathecal antibody synthesis for the specific infectious agent |
| Hestad, 2016 (8) | 44, depression (ICD-10 and DSM-4) | 21 (48)/44.7 | NA | 21, neurological | 7 (33)/47.9 | ↔IL-1β; IL-1 receptor antagonist (IL-1Ra); IL-2; IL-4; IL-5; IL-6; IL-8 (CXCL8); IL-9; IL-10; IL-13; IL-15; IL-17; eotaxin/CCL11; basic fibroblast growth factor; granulocyte macrophage  CSF; interferon (IFN)-γ; IFN-inducible protein 10 (IP-10;  CXCL10); monocyte chemoattractant protein 1 (MCP-1; CCL2); macrophage inflammatory peptide (MIP)-1α (CCL3); MIP-1β (CCL4); platelet-derived growth factor-BB; regulated on activation, normal T-cell expressed and secreted (RANTES; CCL5) tumor necrosis factor alpha; and vascular endothelial growth factor: no difference between cases and controls  IL-7, IL-12 (p70) and granulocyte-colony stimulating factor (G-CSF): not detectable in cases or controls  Correlation between serum and CSF levels of eotaxin, IP-10 and MIP-1 beta  Correlation between IL-15 and MCP-1 and psychiatric symptoms (MADRS, BDI-2) |
| **Combined case groups of psychosis and affective disorders** | | | | | | |
| **Study** | **Case subjects** | | | **Control subjects** | | **Results** |
|  | **N, diagnosis (diagnostic tool)** | **No. (%) of Males/Mean Age, y** | **Medication status** | **N, type** | **No. (%) of Males/Mean Age, y** |  |
| Maxeiner, 2009 (2) | 17, schizophrenia and affective spectrum disorders (ICD-10) | 11 (65)/39.5 | NA | 16, neurological | 9 (56)/54.6 | Cell count: range 1-5/µL in cases and 0-3/µL in controls  ↔Albumin ratio: no difference between cases and controls  Oligoclonal bands in serum or CSF only: not present in cases or controls  Oligoclonal bands present in CSF and serum: in 1 case (5.9%) and 2 controls (12.5%)  ↔CSF/serum ratio of IgG, IgA, IgM: no difference between cases and controls  ↔Frequency of CD4+CD45RO+ and CD8+CD45RO+ cells: no difference between cases (39.85%) and controls (31.30%)  Subset of lymphocytes with>10% CD4+CD127^dim^: present in 6 cases (35.3%) and 3 controls (18.8%) |

Abbreviation: NA: Not Available; MDD: Major Depressive Disorder; DSM: Diagnostics and Statistics Manual; ICD: International Classification of Diseases; PSE: Present Stata Examination; SADS: Schedule for Affective Disorders and Schizophrenia; HAMD: Hamilton Depression score; MADRS: Montgomery and Aasberg Depression Rating Scale; YMRS: Young Mania Rating Scale; CGI: Clinical Global Impression scale; PSAS: Psychiatric Symptom Assessment Scale; BPRS: Brief Psychiatric Rating Scale; PANSS: Positive and Negative Symptom Scale; SAPS: Scale for Assessment of Positive Symptoms; GAF: Global Assessment of Functioning; IL: Interleukin; IFN: Interferon; TNF: Tumor Necrosis Factor; Ig: Immunoglobulin; TGF: Transforming Growth Factor; GM-CSF: granulocyte-macrophage colony-stimulating factor; MIP: Macrophage Inflammatory Protein; MCP: Monocyte Chemoattractant Protein; IP: Inducible Protein; CMV: Cytomegalovirus; HSV: Herpes Simplex Virus.

^i^ The study included both cases with psychosis and affective disorders and is thus shown in the table twice

^ii^ CSF only available from 48 cases

^iii^ Only 19 cases and 20 controls underwent LP

^iv^ Only 9 cases underwent LP

^v^ Only 10 cases and 20 controls underwent LP

^vi^ Same cases as in Levine et al., 1999 (14), but only new results are shown

^vii^ In this study, the MDD group served as controls to the neurological group

| **eTable 3. Baseline characteristics and results from studies without control subjects.** | | | | |
| --- | --- | --- | --- | --- |
| **Schizophrenia spectrum disorders** | | | | |
| **Study** | **N, diagnosis (diagnostic tool)** | **No. (%) of Males/Mean Age, y** | **Medication status** | **Results** |
| Bruetsch, 1942 (47) | 1,281, dementia praecox (NA) | 642 (50)/NA | NA | ↑Cell count in 4/634 cases (0.6%)  ↑Total protein in 85/1,281 cases (6.6%)  Neg. syphilis (Wassermann) |
| ^i^Hunter, 1969 (48) | 256, psychiatric in-patients with various psychiatric diagnoses | 146 (57)/NA | NA | Total protein and gamma-globulin: Abnormal levels in 68 cases, of which 16 cases had schizophrenic syndromes |
| Libíkowa, 1977 (49) | 28, schizophrenia (NA) | NA/NA | All cases received psychotropic medication | Interferon present in 4 cases (14%) and HSV-1 antibodies in 8 cases (29%) |
| Härnryd, 1979 (50) | 12, psychosis (NA) | 0 (0)/40.7 | No psychotropic medication for 2 weeks | Total protein, mean (SD): 380 (33) ng/mL |
| ^ii^Libíková, 1979 (51) | 13, psychosis including both depressive syndromes and mania with psychosis (NA) | 6 (46)/42 | NA | Interferon present in 17/29 (59%) and HSV-1 antibodies in 20/29 cases (69%) |
| ^iii^Libíková, 1979 (52) | 82, schizophrenia (NA) | NA/NA | NA | HSV-1 antibodies present in 43/135 cases (31.9%)  ↓CSF/serum ratio HSV-1 antibodies in 6/107 cases (5.6%)  Pos. tick-borne encephalitic virus test in 9/123 cases (7.3%)  Pos. orbivirus lipovnik antibodies in 1/36 cases (2.8%)  Pos. complement fixation test for orbivirus lipovnik in 1/35 cases (2.9%)  Pos. choriomeningitis virus test in 5/94 cases (5.3%)  Pos. viral antibodies in 39/98 cases (39.8%)  Pos. interferon in 33/98 cases (33.7%) |
| Tyrrell, 1979 (53) | 38, schizophrenia (NA) | NA/NA | NA | Cytopathic effect in 13 cases (34.2%) |
| ^iv^Axelsson, 1982 (54) | 31, psychosis (DSM-3) | 11 (35)/54 | All cases received antipsychotic medication | Impairment of the BBB in 7 cases (24%) of whom 5 (17%) had increased CSF total protein, albumin and IgG. Normal BBB in 18 cases (62%).  ↑Immunoglobulin (isolated) in 1 case (3%)  ↔No correlation between impairment of the BBB and antipsychotic medication or psychiatric scores (CPRS) |
| Mered, 1983 (55) | 23, schizophrenia (research diagnostic criteria NOS) | NA/NA | 6 cases did not receive psychotropic medication > 1 month | ↔No cytopathic effect |
| ^v^Van Kammen, 1984 (56) | 56, schizophrenia (DSM-3) | NA/NA | No psychotropic medication ≥ 2 weeks | ↑CMV IgM in 5/27 (18.5%) |
| Kirch, 1985 (57) | 24, schizophrenia (DSM-3) | 16 (67)/30.1 | 16 cases did not receive antipsychotic medication > 4 weeks | ↑Albumin ratio in 7 cases (29.2%) ↑IgG index in 8 cases (33.3%), of whom 1/8 case (12.5%) had OCB in CSF  ↔No correlation between albumin ratio and antipsychotic medication |
| Torrey, 1985 (58) | 58, schizophrenia and schizoaffective disorder (DSM-3) (group 1)  105, schizophrenia and schizoaffective disorder (NA) (group 2) | 34 (59)/32.5  NA/NA | NA | ↑Total protein in 9/105 cases (9%) (group 2)  ↑Albumin ratio in 4/58 cases (7%) (group 1)  Neg. syphilis |
| Bartova, 1987 (59) | 211, schizophrenia (NA) | NA/NA | NA | Pos. HSV-1 antibodies in 48 cases (18%) by virus neutralization test and in 161 cases (61%) by ELISA |
| Bauer, 1987 (60) | 15, schizophrenia (NA) | 15 (100)/23.6 | 5 cases did not receive antipsychotic medication | Normal cell count and IgG index  ↑Albumin ratio in 5 cases (33%) by reference value of Tibbling et al. and in 8 cases (53%) by reference value of Reiber et al.  ↑IgG in 1 case (6.7%)  Antibodies for rubella, measles, mumps, enterovirus, lymphocytic choriomeningitis, HSV-1+2, VZV, Russian spring summer encephalitis, central European encephalitis not present in 11/11 cases  Syphilis not present in 15/15 cases  ↔No correlation between albumin ratio and antipsychotic medication |
| Tiwari, 1989 (61) | 40, schizophrenia (Diagnostic criteria by Feighner et al.) | NA/31.8 | No psychotropic medication for 15 days apart from pn barbiturates | IgG/total protein%, mean (SE): 6.9 (0.50) in cases with first episode illness, 8.8 (0.82) in cases with second episode illness, 10.3 (0.42) in cases with third episode illness |
| ^i^Samuelsson, 1994 (62) | 125, schizophrenia (Diagnostic criteria by Feighner et al.) | 63 (50)/28.2 | No psychotropic medication but 3 cases had taken sedatives ≥ once prior to admission | Total protein, mean (SD): 31.9 (10.3) mg/dL  Neg. Wasserman/syphilis |
| ^i,vi^Bechter, 1995 (63) | 12, schizophrenia (ICD-10) | NA/NA | Many cases were receiving psychotropic medication | Normal cell count (0-5/mm^3^), total protein  No BBB dysfunction, CSF oligoclonal bands, intrathecal IgG production  ↑Borna disease virus (BDV) index ((CSF BDV-IgG/total CSF-IgG)/(serum BDV-IgG/total serum-IgG)) in 5 cases (41.7%) |
| ^vii^McAllister, 1995 (64) | 79, schizophrenia (DSM-3-R) | 79 (100)/35.1 | Cases were stabilized on haloperidol treatment followed by placebo for 6 weeks or until relapse | IL-1beta present in 2 cases (3%)  ↓IL-1alpha after haloperidol withdrawal (p<0.04) but without correlation to relapse status.  ↔No correlation between IL-2 and haloperidol treatment or between IL-1alpha or IL-2 and psychiatric symptoms (BPRS or SANS)  Correlation between IL-2 and relapse (p<0.05) with higher levels of IL-2 in cases who relapsed in both the haloperidol and drug-free states. |
| Müller, 1995 (65) | 27, schizophrenia (ICD-9) | NA/29 | 12 cases had not received antipsychotic medication ≥ 3 months | ↑Total protein in 9 cases (33.3%)  ↑Albumin, IgG and IgG CSF/serum ratio in 4 cases (14.8%)  ↑Albumin ratio in 6 cases (22.2%)  Correlation between albumin and IgG with psychiatric symptoms (SANS) |
| Mittleman, 1997 (66) | 22, schizophrenia (DMS-3-R) | 12 (55)/14.3 | No psychotropic medication for 4 weeks | IL-2 present in 21/22 cases (95%), IL-4 in 14/22 (64%), IL-5 in 8/20 (40%), IL-10 in 3/22 (14%), IFN-gamma in 3/22 (14%), TNF-beta/LT in 9/22 (41%), and TNF-alpha in 10/20 (50%). |
| Müller, 1997 (6) | 25, schizophrenia (DMS-3-R, ICD-10) | 15 (60)/34 | All cases received antipsychotic medication | Albumin >35 mg% in 4 cases (16%)  BBB disturbance in 10 cases (40%)  ↑IgG in 5 cases (20%)  sIL-6r, mean (SD): 854 (318) pg/mL  ↔No correlation **between** serum and CSF albumin or sIL-6r and negative symptoms (AMDP scale) (p≤0.39)  Correlation between serum and CSF sIL-6r (p≤0.001) |
| Schwarz, 1998 (67) | 40, schizophrenia (DSM-3-R) | 19 (48)/34.98 | All cases received antipsychotic medication | Cell count, mean (SD): 2 (1.63)/μl; Total protein, mean (SD): 41.94 (36.93) mg/dl; Albumin, mean (SD): 23.72 (12.29) mg/dl; Albumin ratio, mean (SD): 6.18 (5.09); BCB impairment in 7 cases (17.5%); IgG, mean (SD): 2.95 (1.85) mg/dl; IgG CSF/serum ratio, mean (SD): 2.81 (1.70); sICAM-1, mean (SD): 0.18 (0.28) ng/ml; Oligoclonal bands not present |
| Yao, 2003 (68) | NA, schizophrenia (DSM-4) | NA/NA | All cases received antipsychotic medication | ↔No correlation between IL-6 or IL-10 and symptoms of psychosis (BHPR, BPRS, SANS) |
| ^i^Brettschneider, 2005 (69) | 41, schizophrenia (ICD-10 and DSM-4) | NA/NA | NA | ↑Albumin ratio (isolated, other CSF parameters normal) in 9 cases (21.9%)  ↔No correlation between albumin ratio and antipsychotic medication (p=0.47). |
| Kranaster, 2011 (70) | 155, schizophrenic or schizophreniform psychosis (DSM-4) | 95 (61)/29.8 | No cases received antipsychotic medication; benzodiazepines were tolerated. | CSF pathologies leading to the rediagnoses in 5 cases (3.2%): HSV-encephalitis, neuroborreliosis, and chronic CNS inflammation of uncertain origin  Mild to moderate adverse events to LP in 16 cases (10.3%) (mostly headache or local pain at the puncture site and two cases of post-LP headache syndrome with nausea) |
| ^viii^Endres, 2015 (71) | 180, schizophreniform syndrome, schizoaffective syndrome, psychotic syndrome in context of other disorders (NA) | 79 (44)/34.67 | NA | ↑Cell count in 6/179 cases (3.4%) (>5 cells/µL)  ↑Total protein in 76/180 cases (42.2%)  ↑Albumin ratio in 39/179 cases (21.8%)  Intrathecal immunoglobulin synthesis in 13/180 cases (7.2%)  Oligoclonal bands restricted to CSF in 10/180 cases (5.6%)  Identical oligoclonal bands in CSF and blood in 3/180 cases (1.7%)  Antibodies against neuronal cell surface antigens:  Anti-VGKC-complex in 3/125 cases (2.4%)  Anti-NMDAR in 1/125 cases (0.8%)  No anti-AMPAR or anti-GABA-B antibodies (0/96)  Antibodies against intracellular synaptic antigens:  No anti-GAD or anti-ampiphysin antibodies (0/142)  Antibodies against intracellular onconeural antigens:  Anti-Yo reactivity in 3/142 cases (2.1%)  Anti-Hu reactivity in 1/142 cases (0.7%) (low positive)  Anti-cv2 (CRMP5) reactivity in 1/142 cases (0.7%) (low positive)  No antibodies with anti-Ri, anti-Ma1-Ma2, or anti-SOX1 reactivity (0/142) |
| **Affective disorders** | | | | |
| **Study** | **N, diagnosis** | **No. (%) of Males/Mean Age, y** | **Medication status** |  |
| ^i^Hunter, 1969 (48) | 256, psychiatric in-patients with various psychiatric diagnoses | 146 (57)/NA | NA | Abnormal levels of total protein and gamma-globulin in 68 cases, of which 17 cases had depressive syndromes |
| ^ix^Niklasson, 1984 (9) | 99, unipolar depression, bipolar disorder and schizoaffective disorder ( Diagnostic criteria by Spitzer et al., 1978) | 41 (41)/41.8 | No antidepressant or antipsychotic medication ≥ 10 days. Benzodiazepines were allowed and continued use of lithium was allowed in 3 cases. | Albumin, mean (SD) males=264.4 (110.7), females=211.4 (77.4) mg/L (p=0.005); Albumin ratio, mean (SD): males=0.0055 (0.0023), females=0.0046 (0.0017) (p=0.07)  ↔No correlation between CSF and serum albumin levels. |
| ^i^Samuelsson, 1994 (62) | 180, MDD  101, bipolar disorder ( Diagnostic criteria by Feighner et al.) | 77 (43) and 39 (39)/43.9 and 35.4 | No psychotropic medication but 32 had taken sedatives ≥ once prior to admission | Protein, mean (SD) mg/dL: MDD=33.5 (14.1); bipolar disorder=30.7 (12.2)  Neg. Wasserman/syphilis  ↔No correlation between total protein in MDD cases and previous sedative medication |
| ^i,vi^Bechter, 1995 (63) | 14, mania, bipolar disorder, and depression (ICD-10) | NA/NA | Many cases received psychotropic medication | Cell count (0-5/mm^3^), total protein: Normal  BBB dysfunction in 3 cases (21.4%)  Oligoclonal bands in CSF in 1 case (7.1%)  No intrathecal IgG production  ↑BDV index in 2 cases (14.3%) ((CSF BDV-IgG/total CSF-IgG)/(serum BDV-IgG/total serum-IgG)) |
| Zachrisson, 2000 (72) | 9, MDD and bipolar disorder type 2 (DSM-5) | 8 (89)/57.6 | 8 cases received psychotropic medication | ↑Albumin ratio in 4 cases (44%) |
| ^i^Brettschneider, 2005 (69) | 100, depression (ICD-10 and DSM-4) | NA/NA | NA | ↑Albumin ratio (isolated, other CSF parameters normal) in 24 cases (24%)  ↔No correlation between albumin ratio and antipsychotic medication (p=0.47). |
| ^viii^Endres, 2016 (73) | 125, depressive syndrome (NA) | 65 (52)/53 | NA | ↑Cell count in 5/125 cases (4%) (>5 cells/µL)  ↑Total protein in 58/125 cases (46.6%)  ↑Albumin ratio in 24/124 cases (19.4%)  ↑IgG index in 4/124 cases (3.2%)  Oligoclonal bands restricted to CSF in 8/124 cases (6.5%)  Identical oligoclonal bands in CSF and blood in 9/124 cases (7.3%)  Antibodies against intracellular neuronal antigens and antibodies against neuronal cell surface antigens investigated in 50.4% and 30.4% of cases, respectively: 1 case (2.6%) pos. for Anti-VGKC antibodies  Correlation between suicidal tendencies and cell count. No other correlations between CSF findings and AMDP or GAF scores. |
| ^x^Endres, 2016 (74) | 60, bipolar disorder (NA) | 25 (40)/48.4 | NA | No antibodies against intracellular antigens or neuronal cell surface antigens |
| **Combined case groups of psychosis and affective disorders** | | | | |
| **Study** | **N, diagnosis** | **No. (%) of Males/Mean Age, y** | **Medication status** |  |
| Dencker, 1961 (75) | 2,161, psychiatric patients (neurasthenia, depression, anxiety state, hysteria, so-called organic defect conditions, psychosis, asocial behavior, debility) | 1135 (53)/NA | NA | Total protein: <20 mg/100mL in 60 cases (3%), ≥70 mg/100mL in 38 cases (1.8%) |
| Taylor, 1985 (76) | 34, schizophrenia and affective disorders (NA) | NA/NA | NA | Virus-like agent present in 23 cases (67.6%) |
| Bechter, 2010 (1) | 63, schizophrenia spectrum disorders and affective spectrum disorders (ICD-10) | NA/39.6 | All cases received psychotropic medication | Abnormal CSF findings in 41% of cases:  ↑Cell count in 6 cases (9.5%) ↑Albumin ratio in 18 cases (29%)  Normal IgG, IgM and IgA  Intrathecal immune response in 9 cases (14%)  Oligoclonal bands restricted to CSF in 4 cases (6%); identical bands in CSF and serum in 5 cases (8%)  CSF pathologies leading to rediagnosis of 4 cases (6%): streptococcal associated autoimmune disorder, late or chronic stage of BDV infection respectively polyspecific intrathecal immunreaction, HSV-1/2 infection associated autoimmune disorder, and undefined systemic autoimmune disorder associated with Sjögren syndrome |
| ^xi^Maxeiner, 2014 (3) | 15, affective disorder  16, schizophrenic disorders  (ICD-10 and DSM-4) | 8 (53)/47.4  13 (81)/36.3 | All cases received psychotropic medication | Marginal elevation of almost every cytokine analyzed (IFN-gamma, TNF-alpha, IL-1beta, IL-2, IL-4, IL-5, IL-8, IL-10, IL12p70, IL-13 and IL-17; data not shown) |

Abbreviation: NA: Not Available; MDD: Major Depressive Disorder; DSM: Diagnostics and Statistics Manual; ICD: International Classification of Diseases; PSE: Present Stata Examination; SADS: Schedule for Affective Disorders and Schizophrenia; HAMD: Hamilton Depression score; MADRS: Montgomery and Aasberg Depression Rating Scale; YMRS: Young Mania Rating Scale; CGI: Clinical Global Impression scale; PSAS: Psychiatric Symptom Assessment Scale; BPRS: Brief Psychiatric Rating Scale; PANSS: Positive and Negative Symptom Scale; SAPS: Scale for Assessment of Positive Symptoms; GAF: Global Assessment of Functioning; IL: Interleukin; IFN: Interferon; TNF: Tumor Necrosis Factor; Ig: Immunoglobulin; TGF: Transforming Growth Factor; GM-CSF: granulocyte-macrophage colony-stimulating factor; MIP: Macrophage Inflammatory Protein; MCP: Monocyte Chemoattractant Protein; IP: Inducible Protein; CMV: Cytomegalovirus; HSV: Herpes Simplex Virus.

^i^ The study included both cases with psychosis and affective disorders and is thus shown in the table twice

^ii^ Giving a total of 29 CSF samples

^iii^ Giving a total of 137 CSF samples

^iv^ 2 cases were excluded due to technicalities regarding sampling

^v^ Only 27 cases tested for IgM

^vi^ Only BDV serum-positive cases were selected for the analyses from a larger group of cases tested for BDV in serum

^vii^ The results are only based on 64 CSF samples

^viii^ Some analyses were performed in a smaller subgroup of the cases

^ix^ Not all cases underwent LP

^x^ Analyses were performed on 63 CSF samples since three cases underwent LP twice. 40 of the cases were also included in Stich et al, 2015, only new results are shown here.

^xi^ Cases were also included in Bechter et al., 2010, only new results are shown here

| **eTable 4. Bias assessment of studies included in the meta-analysis according to the Newcastle-Ottawa quality assessment scale for case control studies.** | | | | | | | | | |
| --- | --- | --- | --- | --- | --- | --- | --- | --- | --- |
| **Psychosis** | | | | | | | | | |
|  | **Selection 1**  **A=** | | **Selection 2**  **A=** | | **Selection 3**  **A=** | **Selection 4**  **A=** | **Comparability 1**  **Age=**  **Other factors=** | **Exposure 1**  **A=**  **B=** | **Exposure 2**  **A=** |
| Roos, 1985 (79) | A | | B | | C | B | 0 stars | 0 stars | A |
| Harrington, 1985 (77) | A | | B | | C | B | 0 stars | 0 stars | A |
| Kirch, 1992 (81) | C | | B | | C | A | 0 stars | 0 stars | B |
| El-Mallakh, 1993 (83) | C | | B | | C | A | 0 stars | 1 star | A |
| Licino, 1993 (84) | A | | B | | C | B | 2 stars (age, gender) | 0 stars | A |
| Katila, 1994 (13) | C | | B | | C | A | 0 stars | 0 stars | A |
| Rapaport, 1997 (82) | C | | B | | A | A | 0 stars | 0 stars | A |
| Vawter, 1997 (91) | C | | B | | C | B | 0 stars | 0 stars | A |
| Van Kammen, 1999 (86) | A | | B | | A | A | 0 stars | 0 stars | A |
| Nikkilä, 2002 (90) | B | | B | | C | A | 0 stars | 0 stars | A |
| Garver, 2003 (87) | C | | B | | C | A | 0 stars | 0 stars | A |
| Bendikov, 2007 (78) | A | | B | | C | B | 0 stars | 0 stars | A |
| Söderlund, 2009 (85) | C | | B | | A | A | 0 stars | 0 stars | A |
| Sasayama, 2013 (15) | A | | B | | A | A | 1 star (age) | 0 stars | A |
| Hayes, 2014 (88) | C | | B | | A | A | 0 stars | 0 stars | A |
| Schwieler, 2015 (89) | A | | A | | A | A | 2 stars (age, BMI) | 0 stars | A |
| Severance, 2015 (80) | C | | B | | A | B | 0 stars | 0 stars | A |
| Coughlin, 2016 (7) | A | | A | | A | A | 0 stars | 0 stars | A |
| **Affective disorders** | | | | | | | | | |
|  | **Selection 1**  **A=** | **Selection 2**  **A=** | | **Selection 3**  **A=** | | **Selection 4**  **A=** | **Comparability 1**  **Age=**  **Other factors=** | **Exposure 1**  **A=**  **B=** | **Exposure 2**  **A=** |
| Pitts, 1990 (92) | C | B | | A | | A | 0 stars | 1 star | A |
| Hampel, 1997 (93) | C | B | | C | | B | 0 stars | 0 stars | A |
| Hampel, 1999 (97) | C | B | | C | | B | 1 star (age) | 0 stars | A |
| Carpenter, 2004 (101) | A | B | | A | | A | 0 stars | 0 stars | A |
| Gudmundsson, 2007 (95) | A | A | | A | | A | 1 star (gender) | 0 stars | A |
| Lindqvist, 2009 (99) | C | B | | C | | A | 1 star (age) | 0 stars | A |
| Pålhagen, 2010 (102) | C | B | | C | | B | 0 stars | 0 stars | A |
| Söderlund, 2011 (100) | A | B | | B | | A | 0 stars | 0 stars | A |
| Martinez, 2012 (98) | A | B | | C | | A | 0 stars | 1 star | A |
| Janelidze, 2013 (104) | A | B | | C | | A | 2 stars (age, gender) | 0 stars | A |
| Kern, 2014 (103) | A | A | | A | | A | 2 stars (age, BMI, smoking) | 0 stars | A |
| Zetterberg, 2014 (96) | A | B | | C | | A | 2 stars (age, gender) | 1 star | A |
| Isgren, 2015 (94) | A | B | | C | | A | 2 stars (age, gender) | 1 star | A |
| Janelidze, 2015 (105) | C | B | | C | | A | 1 star (age) | 0 stars | A |

**eFigure 1: Forest plots on the results from studies investigating CSF immune-related markers in patients with schizophrenia spectrum or affective disorders (i.e. all the remaining forest plots that were not shown in Figure 2 in the main material).**

**Schizophrenia spectrum disorders vs. healthy controls**

**Cell count**


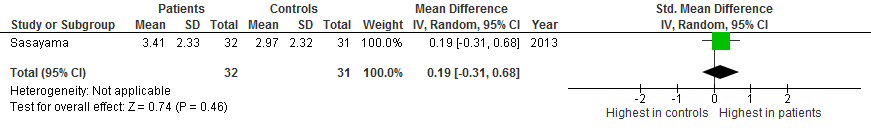


**IgG, mg/dL**

**
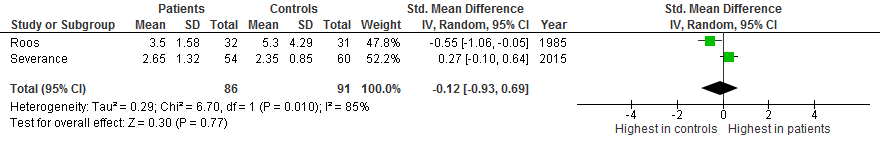
**

**IgG ratio**


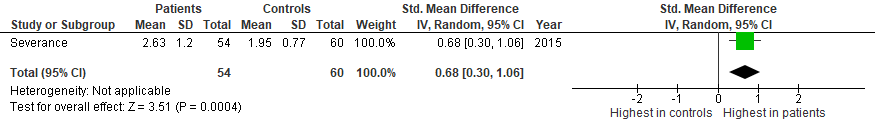


**IgG Albumin ratio**


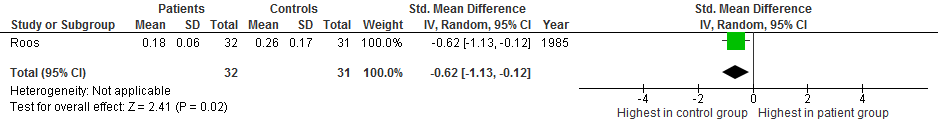


**IgG Index**


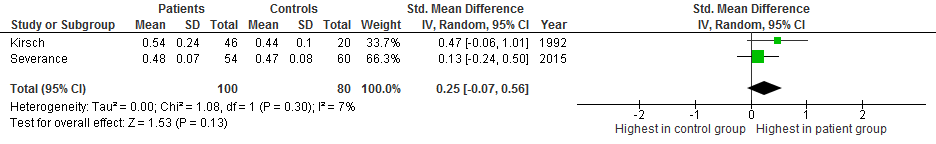


**IL-1 alpha, samples above detection limit, i.e. odds ratio for samples being above detection limit**


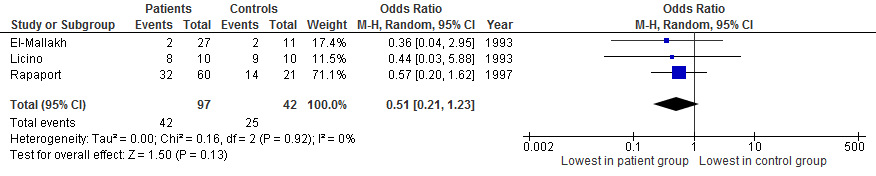


**IL-1 alpha, ng/mL, continuous outcome**


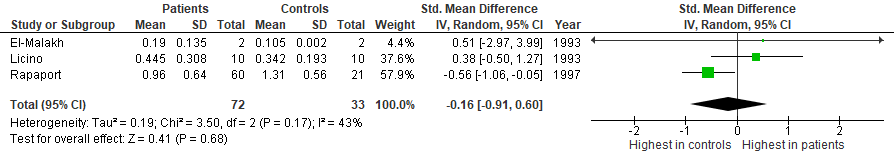


**IL-1 Beta, samples above detection limit, i.e. odds ratio for samples being above detection limit**


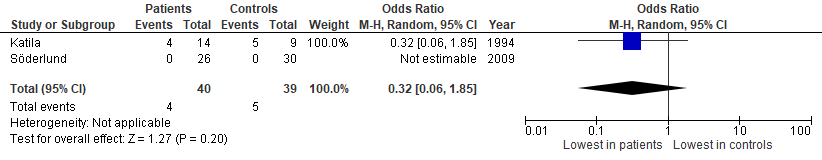


**IL-1 beta, pg/mL, continuous outcome**


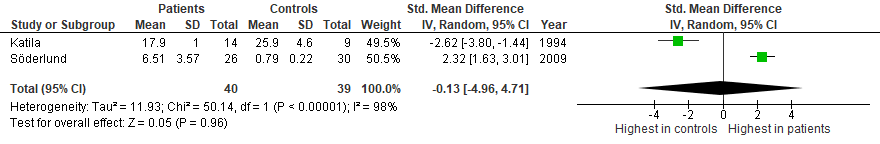


**IL-2, samples above detection limit, i.e. odds ratio for samples being above detection limit**


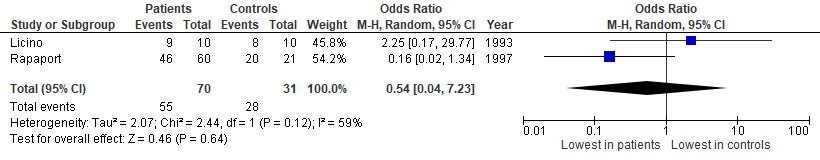


**IL-2, ng/mL, continuous outcome**


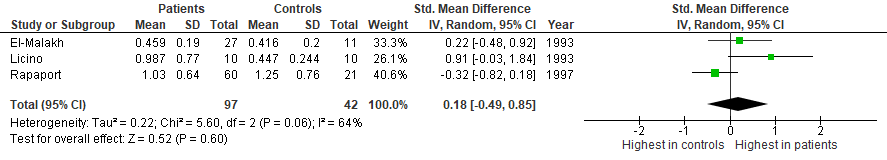


**IL-6, samples above detection limit, i.e. odds ratio for samples being above detection limit**


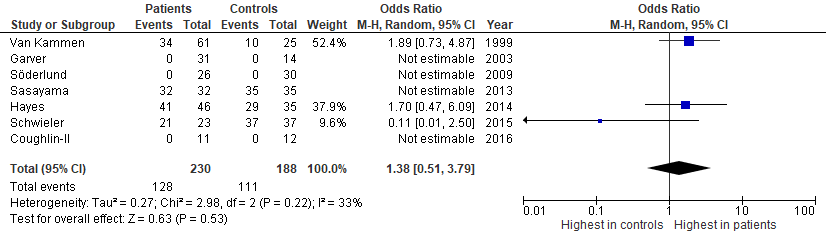


**IL-6R, ng/mL, continuous outcome**


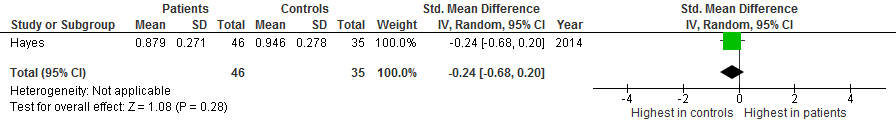


**Neopterin, nmol/L**


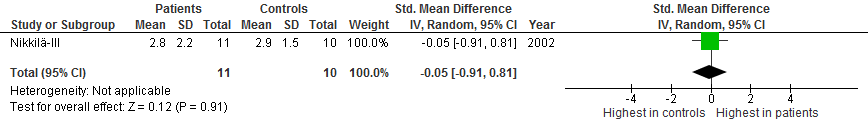


**MIP- 1 alfa, pg/mL**


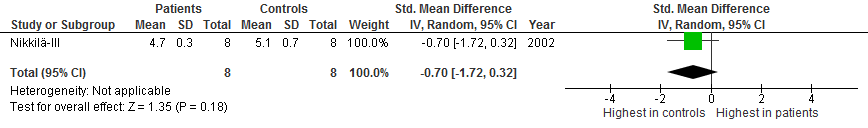


**C3, mg/mL**


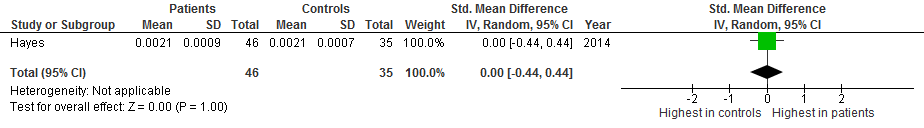


**MCP-2, pg/mL**


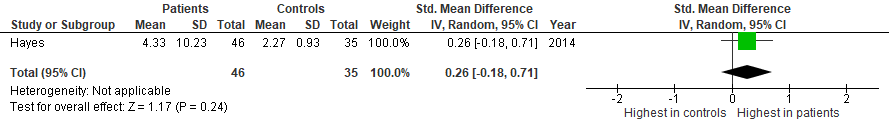


**TNFR2, ng/mL**


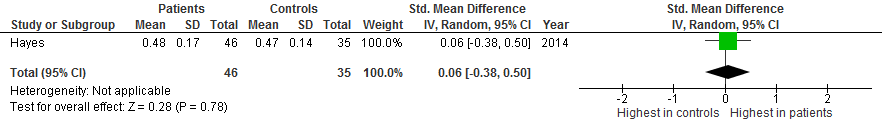


**TGFB1, pg/mL**


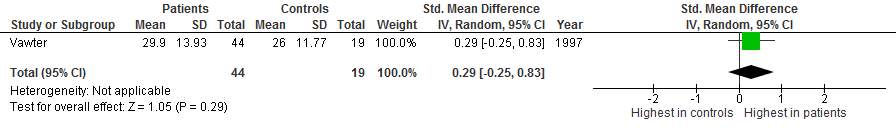


**TGFB2, pg/mL**


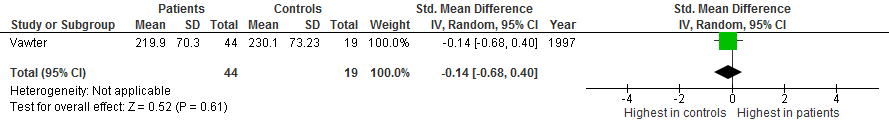


**Affective disorders vs. healthy controls**

**Cell count**


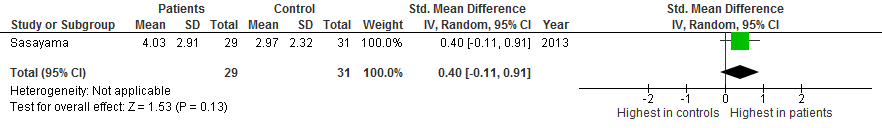


**IgG, mg/dL**


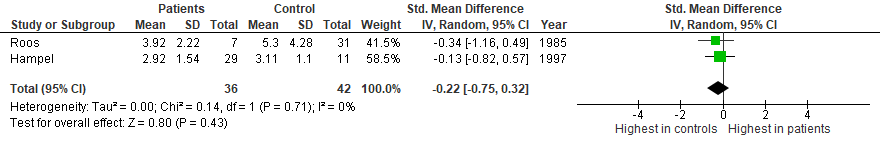


**IgG ratio**


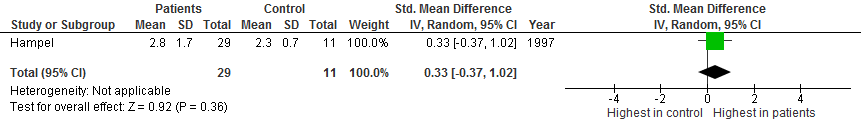


**IgG Albumin ratio**


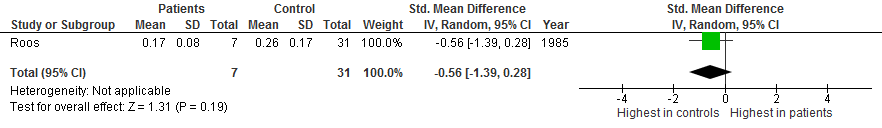


**IgG Index**


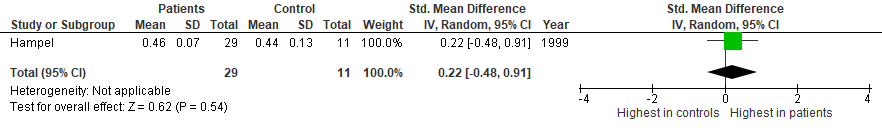


**IL-1, pg/mL**


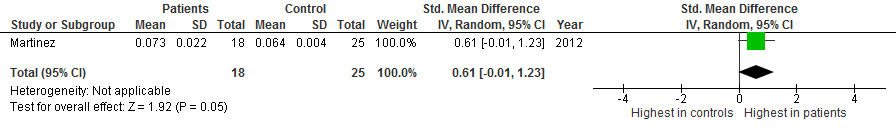


**IL-1 beta, pg/mL**


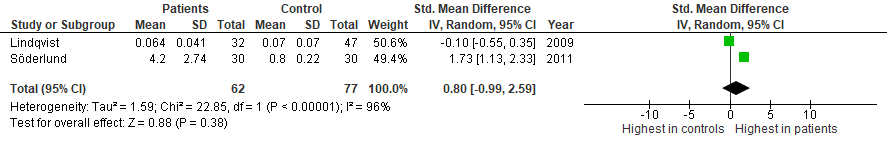


**TNF-alpha, pg/mL**


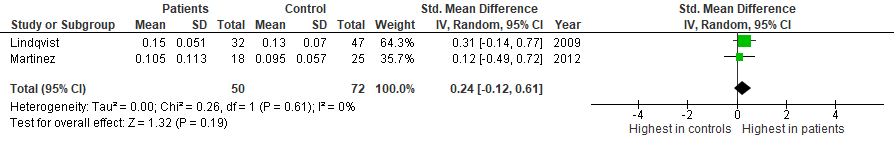


**Eotaxin-1, pg/mL**


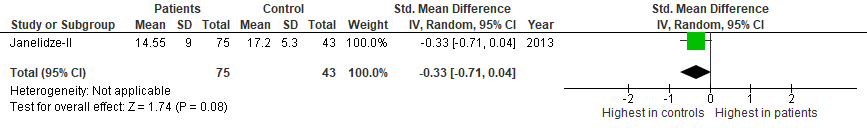


**IP-10, pg/mL**


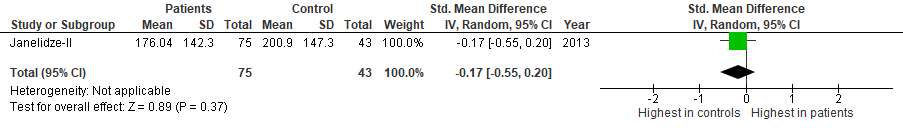


**MIP-1B, pg/mL**


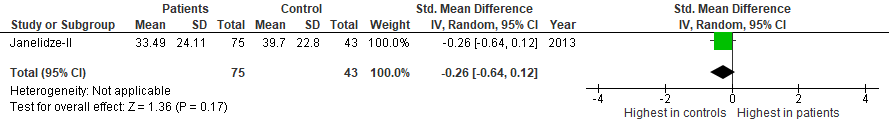


**MCP-1, pg/mL**


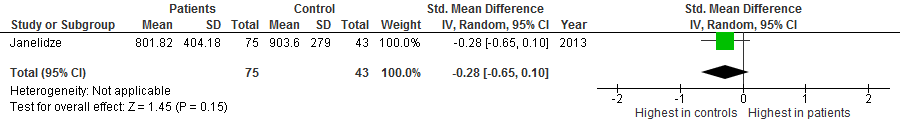


**MCP-4, pg/mL**


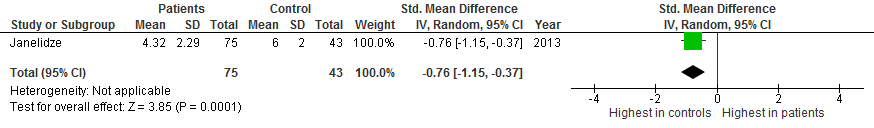


**TARC, pg/mL**


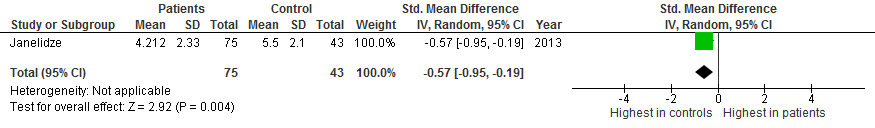


**eFigure 2: Forest plots on the results from studies published after the year 2000 investigating CSF immune-related markers in patients with schizophrenia spectrum or affective disorders.**

**Schizophrenia spectrum disorders vs. healthy controls**

**Total protein, mg/dL**

**
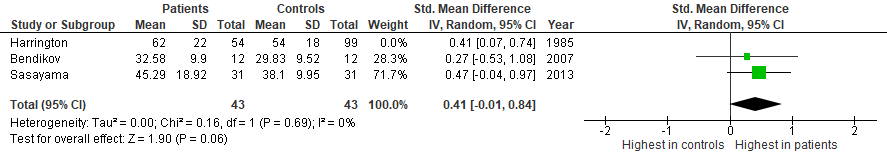
**

**Albumin, mg/dL**


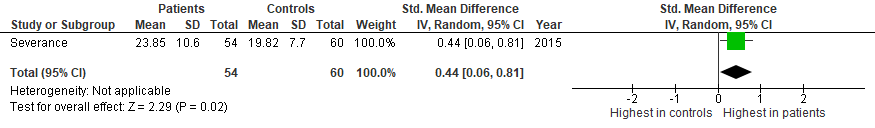


**Albumin ratio**


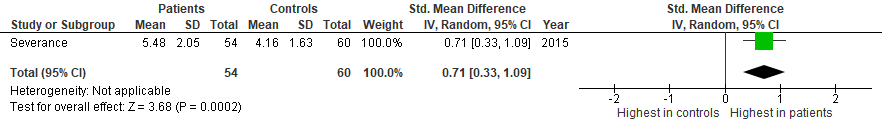


**IL-6, ng/mL**

**
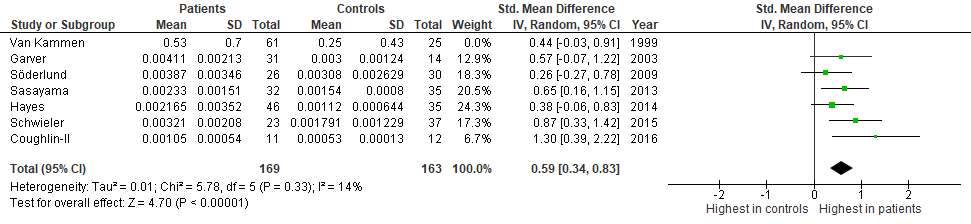
**

**IL-8, pg/mL**


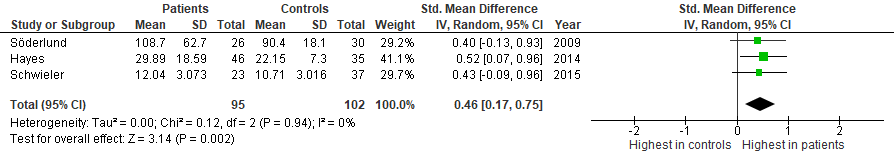


**Cell count**


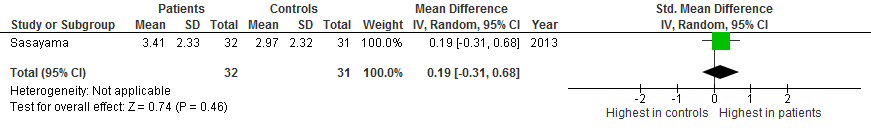


**IgG, mg/dL**


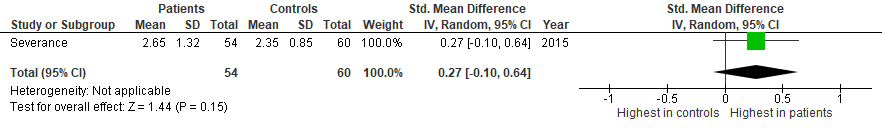


**IgG ratio**


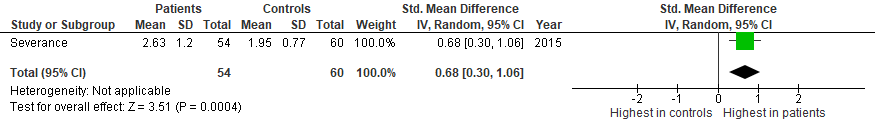


**IgG Index**


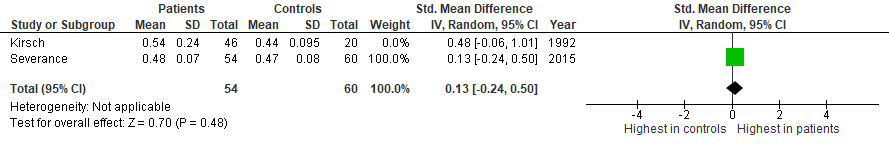


**IL-1 beta, pg/mL, continuous outcome**


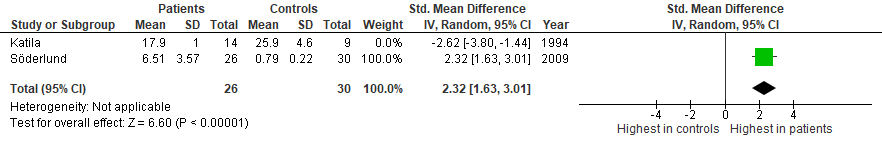


**IL-6, samples above detection limit, i.e. odds ratio for samples being above detection limit**


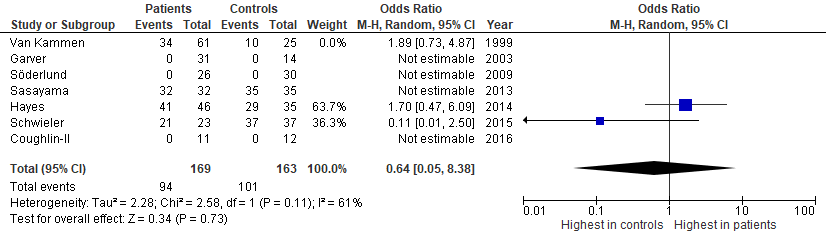


**IL-6R, ng/mL, continuous outcome**


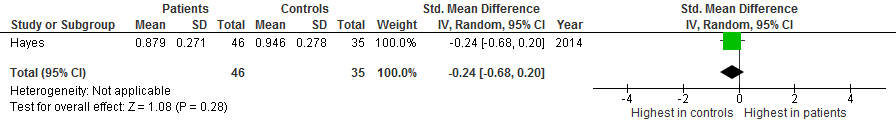


**Neopterin, nmol/L**


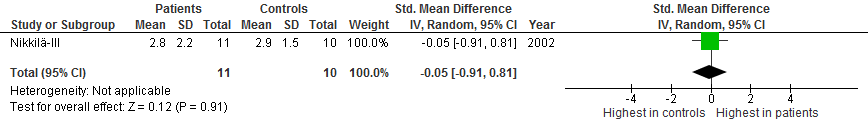


**MIP- 1 alfa, pg/mL**


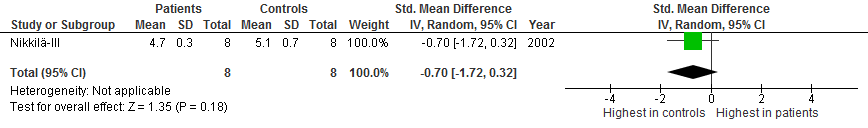


**C3, mg/mL**


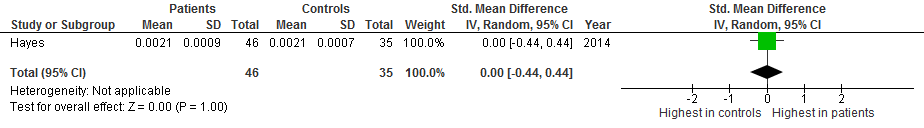


**MCP-2, pg/mL**


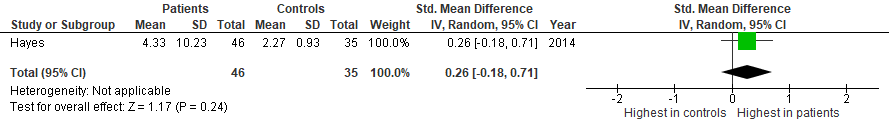


**TNFR2, ng/mL**


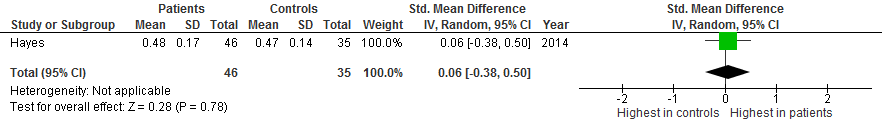


**Affective disorders vs. healthy controls**

**Total protein, mg/dL**


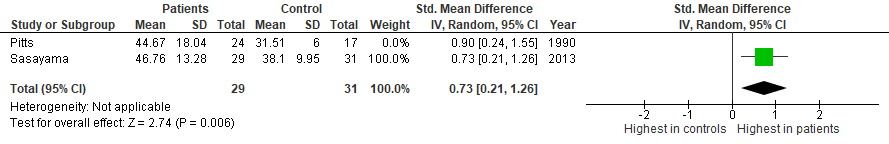


**Albumin, mg/dL**


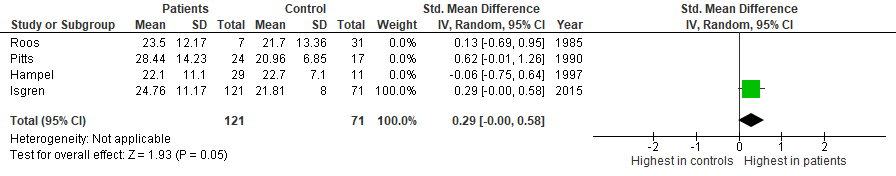


**Albumin ratio**


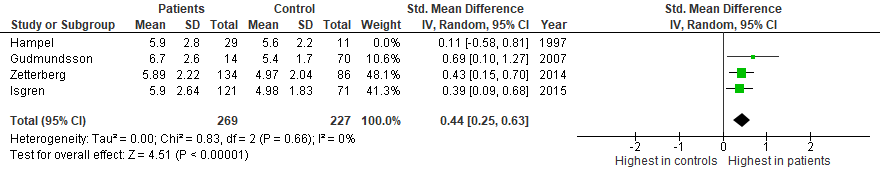


**IL-6, pg/mL**

**
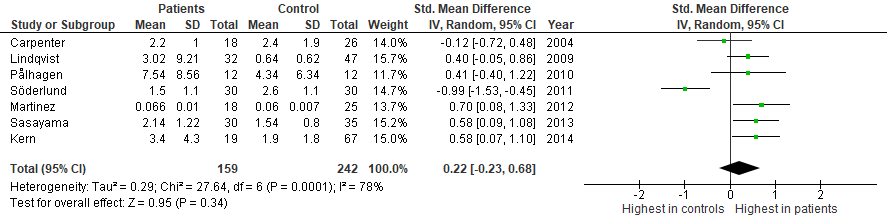
**

**IL-8, pg/mL**


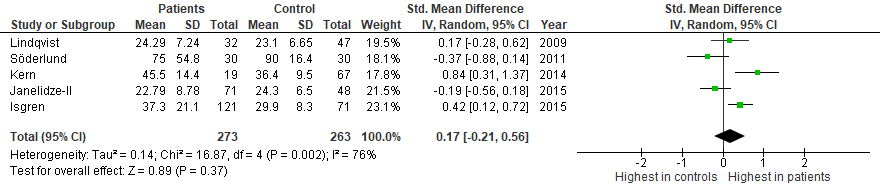


**Cell count**


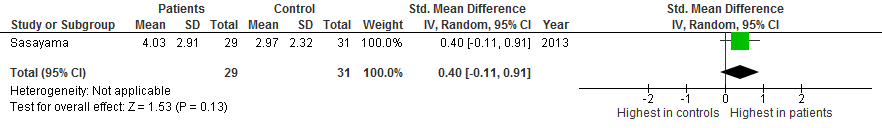


**IL-1, pg/mL**


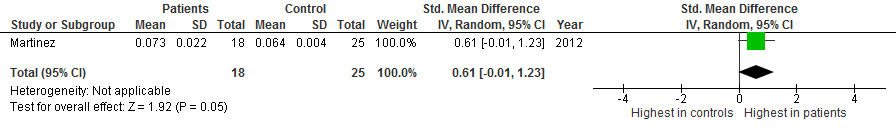


**IL-1 beta, pg/mL**


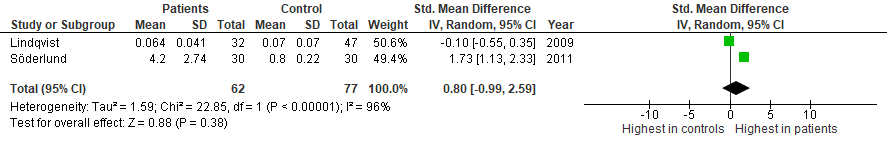


**TNF-alpha, pg/mL**


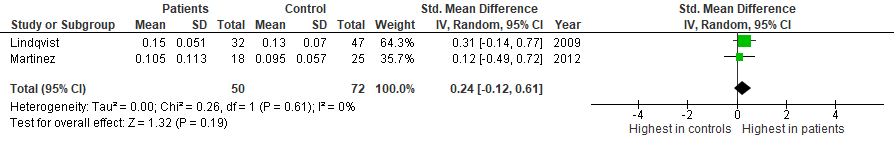


**Eotaxin-1, pg/mL**


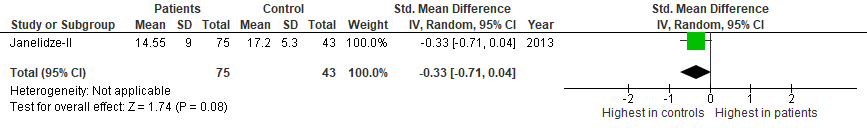


**IP-10, pg/mL**


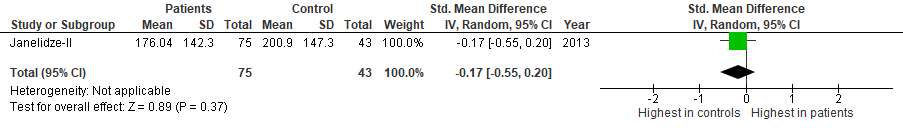


**MIP-1B, pg/mL**


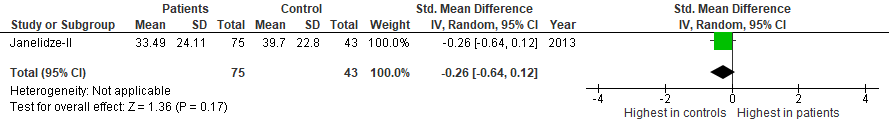


**MCP-1, pg/mL**


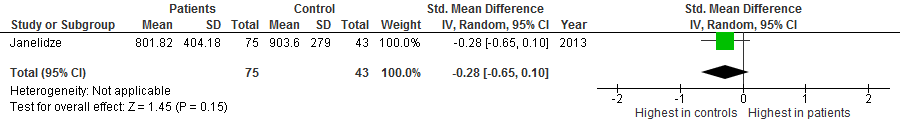


**MCP-4, pg/mL**


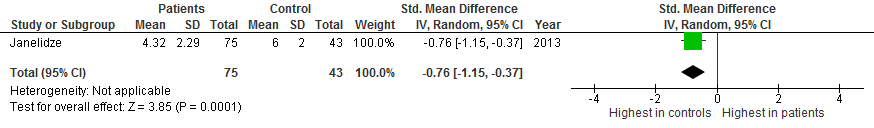


**TARC, pg/mL**


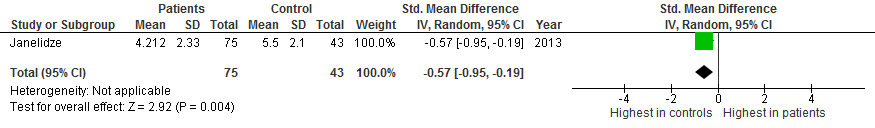


**eFigure 3: Post-hoc analyses comparing patients with acute psychosis or chronic psychosis, which was only possible for IL-6.**

**IL-6, ng/mL:**


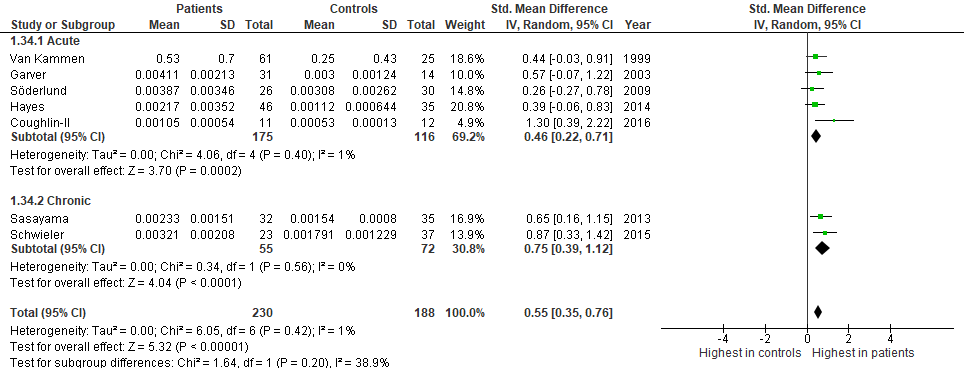


**eReferences**

1. Bechter K, Reiber H, Herzog S, Fuchs D, Tumani H, Maxeiner HG. Cerebrospinal fluid analysis in affective and schizophrenic spectrum disorders: Identification of subgroups with immune responses and blood–CSF barrier dysfunction. J Psychiatr Res [Internet]. Elsevier Ltd; 2010;44(5):321–30. Available from: http://linkinghub.elsevier.com/retrieve/pii/S0022395609001848

2. Maxeiner H-G, Rojewski MT, Schmitt A, Tumani H, Bechter K, Schmitt M. Flow cytometric analysis of T cell subsets in paired samples of cerebrospinal fluid and peripheral blood from patients with neurological and psychiatric disorders. Brain Behav Immun. 2009;23(1):134–42.

3. Maxeiner H-G, Marion Schneider E, Kurfiss S-T, Brettschneider J, Tumani H, Bechter K. Cerebrospinal fluid and serum cytokine profiling to detect immune control of infectious and inflammatory neurological and psychiatric diseases. Cytokine [Internet]. Elsevier Ltd; 2014;69(1):62–7. Available from: http://linkinghub.elsevier.com/retrieve/pii/S1043466614001410

4. Preble OT, Torrey EF. Serum interferon in patients with psychosis. Am J Psychiatry. 1985;142(10):1184–6.

5. Delisi LE, Weinberger DR, Potkin S, Neckers LM, Shiling DJ, Wyatt RJ. Quantitative determination of immunoglobulins in CSF and plasma of chronic schizophrenic patients. Br J Psychiatry. 1981;139(6):513–8.

6. Müller N, Dobmeier P, Empl M, Riedel M, Schwarz M, Ackenheil M. Soluble IL-6 receptors in the serum and cerebrospinal fluid of paranoid schizophrenic patients. Eur Psychiatry. 1997;12(6):294–9.

7. Coughlin JM, Wang Y, Ambinder EB, Ward RE, Minn I, Vranesic M, et al. In vivo markers of inflammatory response in recent-onset schizophrenia: a combined study using [(11)C]DPA-713 PET and analysis of CSF and plasma. Transl Psychiatry [Internet]. 2016 [cited 2016 Nov 18];6:e777. Available from: http://dx.doi.org/10.1038/tp.2016.40

8. Hestad KA, Engedal K, Whist JE, Aukrust P, Farup PG, Mollnes TE, et al. Patients with depression display cytokine levels in serum and cerebrospinal fluid similar to patients with diffuse neurological symptoms without a defined diagnosis. Neuropsychiatr Dis Treat. 2016;12:817–22.

9. Niklasson F, Agren H. Brain energy metabolism and blood-brain barrier permeability in depressive patients: analyses of creatine, creatinine, urate, and albumin in CSF and blood. Biol Psychiatry. 1984;19(8):1183–206.

10. Torrey EF, Peterson MR, Brannon WL, Carpenter WT, Post RM, Van Kammen DP. Immunoglobulins and viral antibodies in psychiatric patients. Br J Psychiatry. 1978;132(4):342–8.

11. Nikkilä H V., Müller K, Ahokas A, Rimón R, Andersson LC. Increased frequency of activated lymphocytes in the cerebrospinal fluid of patients with acute schizophrenia. Schizophr Res. 2001;49(1–2):99–105.

12. Nikkila H, Müller K, Ahokas A, Miettinen K, Andersson LC, Rimón R. Abnormal distributions of T-lymphocyte subsets in the cerebrospinal fluid of patients with acute schizophrenia. Schizophr Res. 1995;14(3):215–21.

13. Katila H, Hurme M, Wahlbeck K, Appelberg B, Rimon R. Plasma and cerebrospinal fluid interleukin-1 beta and interleukin-6 in hospitalized schizophrenic patients. Neuropsychobiology [Internet]. 1994;30(1):20–3. Available from: http://ovidsp.ovid.com/ovidweb.cgi?T=JS&PAGE=reference&D=med3&NEWS=N&AN=7969854

14. Levine J, Barak Y, Chengappa K, Rapoport A, Antelman S, Barak V. Low CSF soluble interleukin 2 receptor levels in acute depression. Short communication. J Neural Transm. 1999;106(9–10):1011–5.

15. Sasayama D, Hattori K, Wakabayashi C, Teraishi T, Hori H, Ota M, et al. Increased cerebrospinal fluid interleukin-6 levels in patients with schizophrenia and those with major depressive disorder. J Psychiatr Res. 2013;47(3):401–6.

16. Hoerster SJ, Hillman F, Bohls S, Lara F, Thurman N. Cerebrospinal fluid in mental diseases (a study using paper electrophoresis). Dis Nerv Syst. 1963;24:357–60.

17. Selecki B, Todd P, Westwood A, Kraus J. Cerebro-spinal fluid and serum protein profiles in deteriorated epileptics, mental defectives with epilepsy, and schizophrenics. Med J Aust. 1964;2:751–3.

18. Shanmugam A. A study of cerebrospinal fluid proteins in schizophrenia. J Indian Med Assoc. 1971;57(6):206–8.

19. Bock E. Immunoglobulins, prealbumin, transferrin, albumin, and alpha2-macroglobulin in cerebrospinal fluid and serum in schizophrenic patients. Birth Defects Orig Artic Ser [Internet]. 1978;14(5):283–95. Available from: NS -

20. Rimon R, Nishmi M, Halonen P. Serum and CSF antibody levels to herpes simplex type 1, measles and rubella viruses in patients with schizophrenia. Ann Clin Res. 1978;10(5):291–3.

21. Crow J, Ferrier I, Johnstone C, MacMillan J, Owens GC, Parry P, et al. Characteristics of patients with schizophrenia or neurological disorder and virus-like agent in cerebrospinal fluid. Lancet. 1979;1(8121):842–4.

22. Albrecht P, Boone E, Torrey EF, Hicks JT, Daniel N. Raised cytomegalovirus-antibody level in cerebrospinal fluid of schizophrenic patients. Lancet. 1980;2(8198):769–72.

23. Gotlieb-Stematsky T, Zonis J, Arlazoroff A, Mozes T, Sigal M, Szekely AG. Antibodies to Epstein-Barr virus, herpes simplex type 1, cytomegalovirus and measles virus in psychiatric patients. Arch Virol. 1981;67(4):333–9.

24. Pandey RS, Gupta AK, Chaturvedi UC. Autoimmune model of schizophrenia with special reference to antibrain antibodies. Biol Psychiatry [Internet]. 1981;16(12):1123–36. Available from: http://www.ncbi.nlm.nih.gov/pubmed/6186298

25. Leonardi A, Cocito L, Tabaton M, Bartolini A, Roccatagliata G. CSF and serum IgG and albumin in schizophrenics. IRCS Med Sci. 1982;10(10):812–3.

26. Torrey E, Yolken R, Winfrey J. Cytomegalovirus Antibody in Cerebrospinal Fluid of Schizophrenic Patients Detected by Enzyme Immunoassay. Science (80- ) [Internet]. 1982;216(4548):892–4. Available from: http://www.jstor.org/stable/1687554%5Cnhttp://about.jstor.org/terms

27. Rimon RH, Halonen P, Lebon P, Heikkilä L, Frey H, Karhula P, et al. Antibrain Antibodies and Interferon in the Serum and the Cerebrospinal Fluid of Patients with Schizophrenia1. Adv Biol psychiatry [Internet]. 1983;12:161–7. Available from: http://www.karger.com/?doi=10.1159/000408324

28. Torrey E, Yolken R, Albrecht P. Cytomegalovirus as a possible etiological agent in schizophrenia. Adv Biol psychiatry. 1983;12:150–60.

29. Tiwari SG, Lal N, Trivedi JK, Sayeed J, Bahauguna LM. Immunoglobulin patterns in schizophrenic patients. Indian J Psychiatry [Internet]. 1984;26(3):223–8. Available from: http://www.ncbi.nlm.nih.gov/pubmed/21965989%5Cnhttp://www.pubmedcentral.nih.gov/articlerender.fcgi?artid=PMC3011242

30. King DJ, Cooper SJ, Earle JAP, Martin SJ, McFerran N V., Wisdom GB. Serum and CSF antibody titres to seven common viruses in schizophrenic patients. Br J Psychiatry. 1985;147(AUG.):145–9.

31. Roy A, Pickar D, Ninan P, Hooks J, Paul SM. A search for interferon in the CSF of chronic schizophrenic patients. Am J Psychiatry. 1985;142(2):269.

32. Shrikhande S, Hirsch SR, Coleman JC, Reveley MA, Dayton R. Cytomegalovirus and schizophrenia. A test of a viral hypothesis. Br J Psychiatry. 1985;146:503–6.

33. Rimon R, Ahokas A, Palo J. Serum and Cerebrospinal Fluid Antibodies To Cytomegalovirus in Schizophrenia. Acta Psychiatr Scand. 1986;73(6):642–4.

34. Bergquist J, Bergquist S, Axelsson R, Ekman R. Demonstration of immunoglobulin G with affinity for dopamine in cerebrospinal fluid from psychotic patients. Clin Chim Acta. 1993;217(2):129–42.

35. Srikanth S, Ravi V, Poornima KS, Shetty KT, Gangadhar BN, Janakiramaiah N. Viral antibodies in recent onset, nonorganic psychoses: Correspondence with symptomatic severity. Biol Psychiatry. 1994;36(8):517–21.

36. Barak V, Barak Y, Levine J, Nisman B, Roisman I. Changes in interleukin-1 beta and soluble interleukin-2 receptor levels in CSF and serum of schizophrenic patients. J Basic Clin Physiol Pharmacol. 1995;6(May 1995):61–9.

37. Sierra-Honigmann AM, Carbone KM, Yolken RH. Polymerase chain reaction (PCR) search for viral nucleic acid sequences in schizophrenia. Br J psychiatry [Internet]. 1995;166(1):55–60. Available from: http://www.ncbi.nlm.nih.gov/pubmed/7894877

38. Deuschle M, Bode L, Heuser I, Schmider J, Ludwig H. Borna disease virus proteins in cerebrospinal fluid of patients with recurrent depression and multiple sclerosis. Lancet [Internet]. 1998;352(9143):1828–9. Available from: http://www.ncbi.nlm.nih.gov/pubmed/12589391

39. Nikkilä H V., Müller K, Ahokas A, Miettinen K, Rimón R, Andersson LC. Accumulation of macrophages in the CSF of schizophrenic patients during acute psychotic episodes. Am J Psychiatry. 1999;156(11):1725–9.

40. Karlsson H, Bachmann S, Schroder J, McArthur J, Torrey EF, Yolken RH. Retroviral RNA identified in the cerebrospinal fluids and brains of individuals with schizophrenia. Proc Natl Acad Sci [Internet]. 2001 [cited 2017 Sep 8];98(8):4634–9. Available from: http://www.pnas.org.ep.fjernadgang.kb.dk/content/98/8/4634.full.pdf

41. Zhu H, Wang D, Liu X. The reduction of CSF tumor necrosis factor alpha levels in schizophrenia: no correlations with psychopathology and coincident metabolic characteristics. Neuropsychiatr Desease Treat. 2016;12:2869–74.

42. Kumar A, Tewari SC, Lal N, Trivedi JK, Bahuguna LM. A study of abnormal CSF total proteins and immunoglobulins levels in patients of depression. Indian J Physiol Pharmacol. 1986;30(1):103–6.

43. Tiwari SG, Lal N, Trivedi JK, Chaturvedi UC, Varma SL, Bahauguna LM. Immunoglobulins and Viral Antibodies in Depressive Patients. Indian J Psychiatry. 1990;32(4):318–23.

44. Levine J, Barak Y, Chengappa K, Rapoport A, Rebey M, Barak V. Cerebrospinal cytokine levels in patients with acute depression. Neuropsychobiology [Internet]. 1999;40(4):171–6. Available from: http://content.karger.com/ProdukteDB/produkte.asp?Aktion=ShowAbstractBuch&ArtikelNr=26615&ProduktNr=226282

45. Schuld A, Uhr M, Pollmächer T. Oligoclonal bands and specific antibody indices in human narcolepsy. Somnologie. 2004;8(3):71–4.

46. Stich O, Andres TA, Gross CM, Gerber SI, Rauer S, Langosch JM. An observational study of inflammation in the central nervous system in patients with bipolar disorder. Bipolar Disord. 2015;17(3):291–302.

47. Bruetsch W, Bahr M, Skobba J, Dieter W. The group of dementia praecox patients with an increase of the protein content of the cerebrospinal fluid. J Nerv Ment Dis. 1942;95(6):669–79.

48. Hunter R, Jones M, Malleson A. Abnormal Cerebrospinal Fluid Total Protein and Gamma-globulin Levels in 256 Patients admitted to a Psychiatric Unit. J Neurol Sci. 1969;9(1):11–38.

49. Libíkowa H, Stancek D, Wiedermann V, Hasto J, Breier S. Psychopharmaca and electroconvulsive therapy in relation to viral antibodies and interferon. Experimental and clinical study. Arch Immunol Ther Exp. 1977;25(5):641–9.

50. Härnryd C, Bjerkenstedt L, Grimm VE, Sedvall G. Reduction of MOPEG levels in cerebrospinal fluid of psychotic women after electroconvulsive treatment. Psychopharmacology (Berl). 1979;64(2):131–4.

51. Libíková H, Pogady J, Rajcani J, Skodacek I, Ciampor F, Kocisova M. Latent herpesvirus hominis 1 in the central nervous system of psychotic patients. Acta Virol [Internet]. 1979;23(3):231–9. Available from: http://ovidsp.ovid.com/ovidweb.cgi?T=JS&PAGE=reference&D=med1&NEWS=N&AN=41436

52. Libíková H, Breier S, Kocisová M, Pogády J, Stünzner D, Ujházyová D. Assay of interferon and viral antibodies in the cerebrospinal fluid in clinical neurology and psychiatry. Acta Biol Med Ger [Internet]. 1979;38(5–6):879–93. Available from: http://www.ncbi.nlm.nih.gov/pubmed/525159

53. Tyrrell DAJ, Parry RP, Crow TJ, Johnstone E, Ferrier IN. Possible virus in schizophrenia and some neurological disorders. Lancet [Internet]. 1979 [cited 2017 Sep 12];1(8121):839–41. Available from: http://ac.els-cdn.com.ep.fjernadgang.kb.dk/S0140673679912613/1-s2.0-S0140673679912613-main.pdf?_tid=2e8437de-977a-11e7-b81a-00000aacb362&acdnat=1505193848_26113cab256436b8c6e1df147db0fe07

54. Axelsson R, Martensson E, Alling C. Impairment of the blood-brain barrier as an aetiological factor in paranoid psychosis. Br J Psychiatry. 1982;141(3):273–81.

55. Mered B, Albrecht P, Torrey EF, Weinberger DR, Potkin SG, Winfrey CJ. Failure to isolate virus from csf of schizophrenics. The Lancet. 1983. p. 919.

56. van Kammen DP, Mann L, Scheinin M, van Kammen WB, Linnoila M. Spinal fluid monoamine metabolites and anti-cytomegalovirus antibodies and brain scan evaluation in schizophrenia. Psychopharmacol Bull [Internet]. 1984;20(3):519–22. Available from: http://www.ncbi.nlm.nih.gov/entrez/query.fcgi?cmd=Retrieve&db=PubMed&dopt=Citation&list_uids=6089252

57. Kirch DG, Kaufmann CA, Papadopoulos NM, Martin B, Weinberger DR. Abnormal cerebrospinal fluid protein indices in schizophrenia. Biol Psychiatry. 1985;20(10):1039–46.

58. Torrey E, Albrecht P, Behr D. Permeability of the blood-brain barrier in psychiatric patients. The American Journal of Psychiatry. 1985. p. 657–8.

59. Bartova L, Rajcani J, Pogady J. Herpes simplex virus antibodies in the cerebrospinal fluid of schizophrenic patients. Acta Virol. 1987;31(5):443–6.

60. Bauer K, Kornhuber J. Blood-cerebrospinal fluid barrier in schizophrenic patients. Eur Arch Psychiatry Neurol Sci. 1987;236(5):257–9.

61. Tiwari SC, Lal N, Trivedi JK, Varma SL. Relationship of immunoglobulins with the number & duration of schizophrenic episodes. Indian J Med Res. 1989;90:229–32.

62. Samuelson SD, Winokur G, Pitts a F. Elevated cerebrospinal fluid protein in men with unipolar or bipolar depression. Biol Psychiatry [Internet]. 1994;35(8):539–44. Available from: http://www.ncbi.nlm.nih.gov/pubmed/8038297

63. Bechter K, Herzog S, Behr W, Schüttler R. Investigations of cerebrospinal fluid in Borna disease virus seropositive psychiatric patients. Eur Psychiatry. 1995;10(5):250–8.

64. McAllister CG, Van Kammen DP, Rehn TJ, Miller AL, Gurklis J, Kelley ME, et al. Increases in CSF levels of interleukin-2 in schizophrenia: Effects of recurrence of psychosis and medication status. Am J Psychiatry. 1995;152(9):1291–7.

65. Müller N, Ackenheil M. Immunoglobulin and albumin content of cerebrospinal fluid in schizophrenic patients: Relationship to negative symptomatology. Schizophr Res. 1995;14(3):223–8.

66. Mittleman BB, Castellanos FX, Jacobsen LK, Rapoport JL, Swedo SE, Shearer GM. Cerebrospinal fluid cytokines in pediatric neuropsychiatric disease. J Immunol [Internet]. 1997 [cited 2016 Nov 30];159(6):2994–9. Available from: http://www.jimmunol.org/content/159/6/2994

67. Schwarz MJ, Ackenheil M, Riedel M, Norbert Müller. Blood-cerebrospinal fluid barrier impairment as indicator for an immune process in schizophrenia. Neurosci Lett. 1998;253(3):201–3.

68. Yao JK, Sistilli CG, Van Kammen DP. Membrane polyunsaturated fatty acids and CSF cytokines in patients with schizophrenia. Prostaglandins Leukot Essent Fat Acids. 2003;69(6):429–36.

69. Brettschneider J, Claus A, Kassubek J, Tumani H. Isolated blood-cerebrospinal fluid barrier dysfunction: Prevalence and associated diseases. J Neurol. 2005;252(9):1067–73.

70. Kranaster L, Koethe D, Hoyer C, Meyer-Lindenberg A, Leweke FM. Cerebrospinal fluid diagnostics in first-episode schizophrenia. Eur Arch Psychiatry Clin Neurosci [Internet]. 2011;261(7):529–30. Available from: http://link.springer.com/10.1007/s00406-011-0193-7

71. Endres D, Perlov E, Baumgartner A, Hottenrott T, Dersch R, Stich O, et al. Immunological findings in psychotic syndromes: a tertiary care hospital’s CSF sample of 180 patients. Front Hum Neurosci [Internet]. 2015 Jan [cited 2016 Jan 22];9:476. Available from: http://www.pubmedcentral.nih.gov/articlerender.fcgi?artid=4564575&tool=pmcentrez&rendertype=abstract

72. Zachrisson OCG, Balldin J, Ekman R, Naesh O, Rosengren L, Ågren H, et al. No evident neuronal damage after electroconvulsive therapy. Psychiatry Res. 2000;96(2):157–65.

73. Endres D, Perlov E, Dersch R, Baumgartner A, Hottenrott T, Berger B, et al. Evidence of cerebrospinal fluid abnormalities in patients with depressive syndromes. J Affect Disord [Internet]. Elsevier; 2016;198:178–84. Available from: http://linkinghub.elsevier.com/retrieve/pii/S0165032716301136

74. Endres D, Dersch R, Hottenrott T, Perlov E, Maier S, van Calker D, et al. Alterations in Cerebrospinal Fluid in Patients with Bipolar Syndromes. Front Psychiatry [Internet]. 2016;7(December):1–8. Available from: http://journal.frontiersin.org/article/10.3389/fpsyt.2016.00194/full

75. Dencker S, Zethraeus S. Sex differences in total protein content of cerebrospinal fluid. Acta Psychiatr Neurol Scand. 1961;36(1):76–82.

76. Taylor GR, Crow TJ, Carter GI, Gamble SJ. Cytopathogenic cerebrospinal fluid from neurological and psychiatric patients. Exp Mol Pathol. 1985;42(2):271–7.

77. Harrington MG, Merril CA, Tone EF. Differences in cerebrospinal fluid proteins between patients with schizophrenia and normal persons. Clin Chem. 1985;31(5):722–6.

78. Bendikov I, Nadri C, Amar S, Panizzutti R, De Miranda J, Wolosker H, et al. A CSF and postmortem brain study of d-serine metabolic parameters in schizophrenia. Schizophr Res. 2007;90(1–3):41–51.

79. Roos R, Davis K, Meltzer H. Immunoglobulin studies in patients with psychiatric diseases. Arch Gen Psychiatry. 1985;42(2):124–8.

80. Severance EG, Gressitt KL, Alaedini A, Rohleder C, Enning F, Bumb JM, et al. IgG dynamics of dietary antigens point to cerebrospinal fluid barrier or flow dysfunction in first-episode schizophrenia. Brain Behav Immun [Internet]. Elsevier Inc.; 2015;44:148–58. Available from: http://dx.doi.org/10.1016/j.bbi.2014.09.009

81. Kirch DG, Alexander RC, Suddath RL, Papadopoulos NM, Kaufmann CA, Daniel DG, et al. Blood-CSF barrier permeability and central nervous system immunoglobulin G in schizophrenia. J Neural Transm. Springer-Verlag; 1992;89(3):219–32.

82. Rapaport MH, McAllister CG, Pickar D, Tamarkin L, Kirch DG, Paul SM. CSF IL-1 and IL-2 in medicated schizophrenic patients and normal volunteers. Schizophr Res. 1997;25(2):123–9.

83. El-Mallakh RS, Suddath RL, Wyatt RJ. Interleukin-1 alpha and interleukin-2 in cerebrospinal fluid of schizophrenic subjects. Prog Neuropsychopharmacol Biol Psychiatry. 1993;17(3):383–91.

84. Licinio J, Seibyl JP, Altemus M, Charney DS, Krystal JH. Elevated CSF levels of interleukin-2 in neuroleptic-free schizophrenic patients. Am J Psychiatry. 1993;150(9):1408–10.

85. Söderlund J, Schröder J, Nordin C, Samuelsson M, Walther-Jallow L, Karlsson H, et al. Activation of brain interleukin-1β in schizophrenia. Mol Psychiatry [Internet]. 2009;14(12):1069–71. Available from: http://www.nature.com/doifinder/10.1038/mp.2009.52

86. Van Kammen DP, McAllister-Sistilli CG, Kelley ME, Gurklis JA, Yao JK. Elevated interleukin-6 in schizophrenia. Psychiatry Res. 1999;87(2–3):129–36.

87. Garver DL, Tamas RL, Holcomb JA. Elevated Interleukin-6 in the Cerebrospinal Fluid of a Previously Delineated Schizophrenia Subtype. Neuropsychopharmacology [Internet]. 2003;28(8):1515–20. Available from: http://www.nature.com/doifinder/10.1038/sj.npp.1300217

88. Hayes LN, Severance EG, Leek JT, Gressitt KL, Rohleder C, Coughlin JM, et al. Inflammatory molecular signature associated with infectious agents in psychosis. Schizophr Bull. 2014;40(5):963–72.

89. Schwieler L, Larsson MK, Skogh E, Kegel ME, Orhan F, Abdelmoaty S, et al. Increased levels of IL-6 in the cerebrospinal fluid of patients with chronic schizophrenia — significance for activation of the kynurenine pathway. J Psychiatry Neurosci [Internet]. 2015;40(2):126–33. Available from: http://jpn.ca/vol40-issue2/40-2-126/

90. Nikkilä H V., Ahokas A, Wahlbeck K, Rimón R, Andersson LC. Neopterin and macrophage inflammatory protein-1alpha in the cerebrospinal fluid of schizophrenic patients: no evidence of intrathecal inflammation. Neuropsychobiology [Internet]. 2002 [cited 2016 Nov 30];46(4):169–72. Available from: www.karger.com

91. Vawter MP, Dillon-Carter O, Issa F, Wyatt RJ, Freed WJ. Transforming growth factors beta 1 and beta 2 in the cerebrospinal fluid of chronic schizophrenic patients. Neuropsychopharmacology [Internet]. 1997 [cited 2017 Sep 13];16(1):83–7. Available from: http://ac.els-cdn.com.ep.fjernadgang.kb.dk/S0893133X96001431/1-s2.0-S0893133X96001431-main.pdf?_tid=7a62b708-988c-11e7-9080-00000aacb35f&acdnat=1505311657_9ed50217b7d6719fed5b737be02343e4

92. Pitts AF, Carroll BT, Gehris TL, Kathol RG, Samuelson SD. Elevated CSF protein in male patients with depression. Biol Psychiatry. 1990;28(7):629–37.

93. Hampel H, Kötter HU, Möller H-J. Blood-Cerebrospinal Fluid Barrier Dysfunction for High Molecular Weight Proteins in Alzheimer Disease and Major Depression. Alzheimer Dis Assoc Disord [Internet]. 1997;11(2):78–87. Available from: http://content.wkhealth.com/linkback/openurl?sid=WKPTLP:landingpage&an=00002093-199706000-00004

94. Isgren A, Jakobsson J, Pålsson E, Ekman CJ, Johansson AGM, Sellgren C, et al. Increased cerebrospinal fluid interleukin-8 in bipolar disorder patients associated with lithium and antipsychotic treatment. Brain Behav Immun [Internet]. Elsevier Inc.; 2015;43:198–204. Available from: http://dx.doi.org/10.1016/j.bbi.2014.10.001

95. Gudmundsson P, Skoog I, Waern M, Blennow K, Pálsson S, Rosengren L, et al. The relationship between cerebrospinal fluid biomarkers and depression in elderly women. Am J Geriatr Psychiatry. 2007;15(10):832–8.

96. Zetterberg H, Jakobsson J, Redsäter M, Andreasson U, Pålsson E, Ekman CJ, et al. Blood-cerebrospinal fluid barrier dysfunction in patients with bipolar disorder in relation to antipsychotic treatment. Psychiatry Res [Internet]. Elsevier; 2014;217(3):143–6. Available from: http://dx.doi.org/10.1016/j.psychres.2014.03.045

97. Hampel H, Kotter HU, Padberg F, Korschenhausen DA, Moller HJ. Oligoclonal bands and blood--cerebrospinal-fluid barrier dysfunction in a subset of patients with Alzheimer disease: comparison with vascular dementia, major depression, and multiple sclerosis. [Internet]. Alzheimer disease and associated disorders. 1999. p. 9–19. Available from: http://ovidsp.ovid.com/ovidweb.cgi?T=JS&PAGE=reference&D=med4&NEWS=N&AN=10192637

98. Martinez JM, Garakani A, Yehuda R, Gorman JM. Proinflammatory and “resiliency” proteins in the CSF of patients with major depression. Depress Anxiety. 2012;29(1):32–8.

99. Lindqvist D, Janelidze S, Hagell P, Erhardt S, Samuelsson M, Minthon L, et al. Interleukin-6 Is Elevated in the Cerebrospinal Fluid of Suicide Attempters and Related to Symptom Severity. Biol Psychiatry [Internet]. Society of Biological Psychiatry; 2009;66(3):287–92. Available from: http://dx.doi.org/10.1016/j.biopsych.2009.01.030

100. Söderlund J, Olsson SK, Samuelsson M, Walther-Jallow L, Johansson C, Erhardt S, et al. Elevation of cerebrospinal fluid interleukin-1β in bipolar disorder. J Psychiatry Neurosci. 2011;36(2):114–8.

101. Carpenter LL, Heninger GR, Malison RT, Tyrka AR, Price LH. Cerebrospinal fluid interleukin (IL)-6 in unipolar major depression. J Affect Disord. 2004;79(1–3):285–9.

102. Pålhagen S, Qi H, Mårtensson B, Wålinder J, Granérus AK, Svenningsson P. Monoamines, BDNF, IL-6 and corticosterone in CSF in patients with Parkinson’s disease and major depression. J Neurol [Internet]. 2010 [cited 2017 Sep 20];257(4):524–32. Available from: https://link-springer-com.ep.fjernadgang.kb.dk/content/pdf/10.1007%2Fs00415-009-5353-6.pdf

103. Kern S, Skoog I, Börjesson-Hanson A, Blennow K, Zetterberg H, Östling S, et al. Higher CSF interleukin-6 and CSF interleukin-8 in current depression in older women. Results from a population-based sample. Brain Behav Immun [Internet]. Elsevier Inc.; 2014;41(1):55–8. Available from: http://dx.doi.org/10.1016/j.bbi.2014.05.006

104. Janelidze S, Ventorp F, Erhardt S, Hansson O, Minthon L, Flax J, et al. Altered chemokine levels in the cerebrospinal fluid and plasma of suicide attempters. Psychoneuroendocrinology [Internet]. Elsevier Ltd; 2013;38(6):853–62. Available from: http://dx.doi.org/10.1016/j.psyneuen.2012.09.010

105. Janelidze S, Suchankova P, Ekman A, Erhardt S, Sellgren C, Samuelsson M, et al. Low IL-8 is associated with anxiety in suicidal patients: Genetic variation and decreased protein levels. Acta Psychiatr Scand. 2015;131(4):269–78.
